# Acupuncture for vascular dementia: an overview of systematic reviews and meta-analysis

**DOI:** 10.3389/fnins.2026.1796611

**Published:** 2026-10-19

**Authors:** Yihao Chen, Yuqing Fan, Yichun Wang, Xin Li, Huize Lin, Xue Li, Xiaorong Tang, Nenggui Xu, Zhennan Wu

**Affiliations:** 1 South China Research Center for Acupuncture and Moxibustion, Medical College of Acu-Moxi and Rehabilitation, Guangzhou University of Chinese Medicine, Guangzhou, China; 2 Department of Physiology, University of Auckland, Auckland, New Zealand; 3 Monash Neuromodulation Research Unit, Department of Physiotherapy, School of Primary and Allied Health Care, Faculty of Medicine, Nursing and Health Science, Monash University, Melbourne, VIC, Australia

**Keywords:** acupuncture, data mining, meta-analysis, methodological quality, overview, systematic review, vascular cognitive impairment, vascular dementia

## Abstract

**Objective:**

To systematically synthesize evidence from systematic reviews (SRs) and meta-analyses (MAs) regarding the efficacy and safety of acupuncture for vascular dementia (VD), evaluate the methodological and evidence quality, and characterize commonly used acupoint selection patterns through data mining.

**Methods:**

Eight databases were searched from inception to October 2025. SRs/MAs of randomized controlled trials (RCTs) on acupuncture for VD were included. Methodological quality, completeness of reporting, and certainty of evidence were assessed using AMSTAR-2, PRISMA 2020, and GRADE, respectively. Furthermore, a secondary data mining analysis was conducted on primary RCTs to identify meridian and acupoint selection laws.

**Results:**

Sixteen SRs/MAs were included. According to AMSTAR-2, only one review was of high quality, while 87.5% were rated low or critically low, primarily due to the lack of protocol registration. GRADE assessment indicated that 97.9% of outcomes were of low or very low certainty, mainly downgraded for risk of bias and imprecision. Most pooled estimates favored acupuncture for cognitive and functional outcomes, but certainty of evidence was predominantly low or very low according to GRADE criteria. A frequency analysis of the 242 unique RCTs identified the Governor Vessel (18.8% of meridian usage) and the acupoints GV20 (Baihui), EX-HN1 (Sishencong), and ST36 (Zusanli) among the most commonly used points; these patterns should be considered hypothesis-generating rather than validated treatment recommendations.

**Conclusion:**

Current evidence indicates potential signals of benefit for acupuncture in VD, but findings remain inconclusive owing to prevalent methodological flaws and high heterogeneity in the primary trials. Future research should prioritize rigorous, large-scale, sham-controlled RCTs testing standardized candidate protocols to establish definitive evidence.

**Systematic review registration:**

https://www.crd.york.ac.uk/PROSPERO/view/CRD420251180848, identifier CRD420251180848.

## 1 Introduction

Vascular dementia (VD) is a severe cognitive impairment syndrome caused by cerebrovascular diseases. Its main clinical manifestations include decline in learning and memory abilities, executive dysfunction, and emotional disturbances (Kalaria, 2018; Qi et al., 2024; Zhang X. et al., 2023). VD accounts for approximately 20% of all dementia cases globally, and this proportion reaches about 26% among the Chinese population aged 60 and above. Its incidence is second only to Alzheimer’s disease, making it the second most common cause of dementia (Jia et al., 2020; Wolters and Ikram, 2019; Xu et al., 2019). With the accelerating aging of the population, the prevalence of VD in China continues to rise, severely affecting patients’ quality of life and imposing a substantial caregiving and economic burden on society (Yu and Wang, 2020).

Currently, there is no specific curative therapy for VD in clinical practice. Treatment is primarily divided into pharmacological and non-pharmacological approaches. Pharmacological interventions include agents to improve cerebral microcirculation (e.g., nimodipine), cholinesterase inhibitors (e.g., donepezil), and NMDA receptor antagonists (e.g., memantine) (Cao and Wu, 2025; Ju and Zhang, 2025; McShane et al., 2019). Non-pharmacological therapies, such as rehabilitation training and hyperbaric oxygen therapy, serve as adjuvant measures to help patients maintain functional abilities to some extent (Barha et al., 2019; Li et al., 2021; Liu-Ambrose et al., 2016). However, pharmacological treatments are limited by modest efficacy, potential adverse reactions, and relatively high costs (Hu et al., 2021; Zhang et al., 2018).

In this context, acupuncture has been investigated as a non-pharmacological adjunct for VD, but its effectiveness and safety remain uncertain because of limitations in the current evidence base.

In recent years, clinical research on acupuncture for VD has increased, accompanied by a substantial emergence of related systematic reviews (SRs) and meta-analyses (MAs). Most of these studies conclude that acupuncture is effective in improving cognitive function and activities of daily living in VD patients. However, notable heterogeneity exists among these published SRs/MAs regarding study quality, inclusion criteria, details of interventions, and outcome measures. For example, some reviews include primary clinical trials of low quality with a high risk of bias; variations in acupoint selection, manipulation techniques, treatment frequency, and course duration across studies contribute to considerable clinical heterogeneity. These factors collectively lead to inconsistent conclusions among different SRs/MAs, thereby undermining the reliability of the existing evidence and its strength for guiding clinical practice.

Although several higher-level evidence syntheses, such as overviews or umbrella reviews, have attempted to summarize acupuncture for cognitive impairment or various types of dementia, they exhibit substantial limitations that restrict their clinical utility for VD specifically. Some prior overviews pooled VD together with Alzheimer’s disease or mild cognitive impairment, failing to provide disease-specific, granulate evidence for VD (Lan et al., 2020). Others focused strictly on a single acupuncture modality (e.g., scalp acupuncture or electroacupuncture), leaving the comparative landscape of diverse acupuncture techniques unexplored (Chen et al., 2022). Furthermore, earlier syntheses have become outdated and lack rigorous assessment of overlapping primary trials across included SRs/MAs, which could potentially distort the pooled effect size. While recent network meta-analyses have begun to explore the comparative benefits of combining different acupuncture modalities with conventional treatments for vascular cognitive impairment (VCI; Liao Y. L. et al., 2025), a comprehensive overview that systematically evaluates the overall methodological quality and overlapping data across these individual reviews remains absent.

It is noteworthy that to date, mainstream clinical practice guidelines for VD have not incorporated acupuncture as a recommended adjuvant or alternative therapy. This is primarily due to a lack of evidence characterized by rigorous methodology and definitive conclusions, highlighting a critical gap between the growing body of research and its translation into clinical recommendations.

Therefore, there is a pressing need for a higher-level, more objective, and comprehensive evidence synthesis to clarify the overall research landscape. To address this, our study employs the methodology of an “overview of systematic reviews.” To our knowledge, this study is among the first disease-focused overviews to comprehensively synthesize evidence across all acupuncture modalities specifically for VD. It critically addresses previous limitations by implementing a rigorous overlap analysis (Corrected Covered Area, CCA) to avoid double-counting primary data, and by tracking the latest SRs/MAs up to the present. By re-synthesizing and analyzing the current highest level of evidence, this overview aims to comprehensively assess the overall efficacy and safety of acupuncture for VD, delineate the strengths and limitations of the existing evidence, identify and explore reasons for conflicting conclusions between studies, and ultimately provide more reliable and clearer evidence-based medical support for the clinical application of acupuncture therapy, while also guiding directions for future high-quality clinical research.

## 2 Materials and methods

The methodology of this overview follows the Cochrane Handbook, and the report of this overview is in line with the Preferred Reporting Items for Systematic Reviews and Meta-Analyses (PRISMA) 2020 checklist. This overview has been registered with the PROSPERO website (Registration number: PROSPERO CRD420251180848).

### 2.1 Inclusion and exclusion criteria

To ensure a rigorous and transparent selection process, the eligibility criteria for including systematic reviews and meta-analyses (SRs/MAs) in this overview were established based on the standardized PICOS framework as follows:

(1) Population (P): Patients with a clear and established clinical diagnosis of VD, regardless of their age, gender, ethnicity, nationality, or specific stage of the disease. Systematic reviews that enrolled broader vascular cognitive impairment (VCI) populations were included only if the primary evidence base predominantly represented established VD; the distribution of VD versus broader VCI populations across the included reviews was quantified to ensure disease-specific interpretability. No restrictions were applied regarding the underlying cerebrovascular pathology (e.g., small vessel disease or large vessel infarction), the specific diseased brain areas, or the affected limbs.

(2) Intervention (I): The intervention evaluated in the treatment group of the included SRs/MAs must be acupuncture (including manual acupuncture, electroacupuncture, scalp acupuncture, etc.,) used either as a standalone monotherapy or in combination with other conventional treatments.

(3) Comparator (C): The control group must have received non-acupuncture therapies, such as conventional pharmaceuticals, routine post-stroke care, or sham/placebo acupuncture controls.

(4) Outcomes (O): The outcome measures were classified into primary and secondary outcomes. The primary outcomes included the Mini-Mental State Examination (MMSE) and the Montreal Cognitive Assessment (MoCA) which directly reflect core cognitive improvements. The secondary outcomes included the Hierarchic Dementia Scale (HDS), Activities of Daily Living Scale (ADL Scale), Adverse events, Scale for the differentiation of syndromes of vascular dementia (SDSVD), NIH stroke scale (NIHSS), Total Effective Rate, Clinical Efficacy, Blessed behavior scale (BBS), Activities of Daily Living - Revised (ADL-R), Functional Activities Questionnaire (FAQ), Global Function, Markedly Effective Rate, Barthel ADL Index (BI), Hcy level, CRP level, and TCM syndrome manifestations.

(5) Study Design (S): Eligible studies were limited to systematic reviews and/or meta-analyses that exclusively aggregated primary randomized controlled trials (RCTs). The publication language was restricted to Chinese or English.

### 2.2 Search strategy

A comprehensive computer-based literature search was conducted across eight biomedical databases: China National Knowledge Network (CNKI), Wanfang Database, VIP Database, Chinese Biomedical Literature Database (CBM), PubMed, Embase, Web of Science, and the Cochrane Library, from their inception to October 31, 2025. In addition, gray literature and clinical trial registries–including ClinicalTrials.gov, the Chinese Clinical Trial Registry (ChiCTR), the CNKI Doctoral and Master’s Dissertations Database, and conference proceedings–were manually screened on October 31, 2025, using free-text terms (“acupuncture” AND “vascular dementia”) to identify additional eligible reviews. The retrieval method adopts the combination of subject words and free words. Key words include acupuncture, acupuncture therapy, acupuncture, ear, acupuncture points, vascular dementias, arteriosclerotic dementia, binswanger disease, chronic progressive subcortical encephalopathy, meta-analysis, systematic review. Taking CNKI as an example, the specific search strategy is as follows: (TKA: Vascular dementia + “Vascular dementia (VD)” + “Vascular dementia (VaD)” + Vascular dementia disease + Treatment of vascular dementia (Exact)) OR (TKA: Dementia, Vascular (Exact)) OR (TKA: Arteriosclerotic dementia (Exact)) OR (TKA: Subcortical arteriosclerotic encephalopathy (Exact)) OR (TKA: Binswanger + Binswanger’s disease + Binswanger’s encephalopathy + Binswanger disease (Exact)) OR (TKA: Chronic progressive cortical encephalopathy (Exact)) OR (TKA: Subcortical leukoencephalopathy (Exact)) OR (TKA: Acute-onset vascular dementia (Exact)) OR (TKA: Subcortical vascular dementia (Exact)) AND (TKA: Acupuncture + Acupuncture treatment + Acupuncture therapy + Acupuncture method + Acupuncture points (Exact)) OR (TKA: Acupuncture-moxibustion therapy + “Acupuncture-moxibustion therapy/methods” + “Acupuncture-moxibustion therapy/electroacupuncture” + Warm needle moxibustion therapy (Exact)) OR (TKA: Electroacupuncture + Electroacupuncture treatment + Electroacupuncture therapy + Electroacupuncture stimulation + Electroacupuncture points (Exact)) OR (TKA: Needling method (Exact)) OR (TKA: Acupuncture and moxibustion + Acupuncture-moxibustion treatment (Exact)) AND (TKA: Systematic review + Systematic review methods + Systematic review and meta-analysis + “Systematic review/meta-analysis” + Systematic review with meta-analysis + Systematic review study (Exact)) OR (TKA: Systematic review (Topic) (Exact)) OR (TKA: Meta-analysis (Exact)) OR (TKA: Meta-analysis (Topic) (Exact)) OR (TKA: meta-analysis (Exact)) OR (TKA: Systematic review and meta-analysis (Exact)). The search strategy for the PubMed database is provided in Supplementary File 1.

### 2.3 Literature screening and data extraction

Two researchers independently screened and extracted the literatures, and cross-checked them. Any disagreement during this process were subject to discussion and negotiation or the decision of a third expert. The extracted literature information included: Authors, publication year, nationality, sample size, intervention measures, quality assessment tools and main conclusions.

### 2.4 Quality assessment

Two researchers independently assessed the methodological and evidence quality of the included SRs/MAs, Any discrepancies were resolved by consensus or adjudication by a third author.

#### 2.4.1 Methodological quality assessment

The methodological quality of the included SRs/MAs was evaluated using the Assessment System for Evaluating Methodological Quality 2 (AMSTAR-2). The AMSTAR-2 scale contains 16 items that can be answered with a “yes,” “partially yes” or “no.” Item 1: Whether the research question and inclusion criteria include the elements of PICO; 2: Whether the study method is determined before its implementation, Whether the report is consistent with the plan; 3: Whether to explain the reasons for the study type; 4: Whether the search literature is comprehensive; 5: Whether the literature is independently screened by two people; 6: Whether the data is extracted by two people; 7: Whether to provide the list of documents and reasons for exclusion; 8: Whether to describe the basic characteristics of the included study in detail; 9: Whether the risk of included study bias is reasonably assessed; 10: Whether to report the source of research funding; 11: whether appropriate statistical methods are used to combine and analyze the results during the meta-analysis; 12: whether to consider the risk of bias in the meta-analysis or other evidence integration; 13: Whether the inclusion risk of study bias was considered when interpreting the results; 14: Whether the heterogeneous results are interpreted or discussed; 15: whether the publication bias was investigated during the quantitative synthesis, Whether to discuss its impact on the results; 16: Whether to report conflicts of interest and sources of funding.

According to the AMSTAR-2 evaluation framework, overall confidence in the results of each review was categorized as “high,” “moderate,” “low,” or “critically low.” Ratings were assigned according to the standard AMSTAR-2 algorithm on the basis of the presence of critical weaknesses (Items 2, 4, 7, 9, 11, 13, and 15) and non-critical weaknesses, rather than by calculating a cumulative numerical score.

#### 2.4.2 Completeness of reporting assessment

SR/MA is an important evidence to guide clinical practice. The clarity of its report affects the realization of its clinical value. Standard reports can reduce the bias between actual research results and published results, and increase the transparency of articles. The PRISMA Report Guide is designed to help authors improve the quality of their reports, obtain key information, and improve readability and credibility. The completeness of reporting of the included SRs/MAs was characterized using the PRISMA 2020 checklist. PRISMA 2020 is a reporting guideline rather than a validated instrument of methodological quality, and the assessment is therefore reported as the completeness of reporting rather than as a quality score. Each of the 27 items was scored according to prespecified operational definitions: an item was scored “yes” only when it was reported in sufficient detail to be reproduced or verified without contacting the authors, “partially yes” when it was mentioned but at least one element required for verification or reproduction was missing, and “no” when it was not reported anywhere in the review, including its text, tables, figures, and Supplementary files. Full operational definitions and item-level decision rules are provided in the Supplementary material.

#### 2.4.3 Evidence quality assessment

In order to be useful to decision-makers, clinicians and patients, the SRs/MAs must provide not only the effect estimates of each result, but also the information needed to judge the correctness of these effect estimates. The Grading of Recommendations Assessment, Development, and Evaluation (GRADE; Chen et al., 2022) provides a structure for SRs/MAs and clinical practice guidelines to ensure that it addresses all key issues of outcome evidence quality evaluation related to a specific issue in a consistent and systematic manner. The quality of evidence for each SR/MA outcome was evaluated by the GRADE system. Since the initial quality of evidence for RCTs is high, the quality of evidence for the outcomes of the study was evaluated based on downgrading factors such as limitations, inconsistencies, indirectness, imprecision, and publication bias of the study. According to the level of downgrade, they are rated as “high,” “moderate,” “low” and “very low.”

#### 2.4.4 Data mining and analysis

To further explore the acupoint selection patterns for acupuncture treatment of vascular dementia (VD), a secondary data mining analysis was conducted. To maintain consistency with the evidence synthesized in our overview, all primary RCTs included in the 16 selected SRs/MAs were eligible for inclusion.

First, two reviewers extracted detailed information of the primary RCTs from all included SRs/MAs. A total of 318 primary-study occurrences were initially retrieved (counting each trial once for every review that included it). Subsequently, a database of primary literature was established, and strict deduplication was performed based on the first author, publication year, and title, resulting in 242 unique RCTs. During the data extraction process, 3 RCTs were excluded because their full texts were unavailable, making it impossible to extract the specific acupoint prescriptions. Ultimately, 239 unique RCTs with complete prescription data were included in the final data mining database.

For these 239 RCTs, information on the main acupoints used in the acupuncture prescriptions was extracted. A database of acupuncture prescriptions was constructed using Microsoft Excel 2019. In this study, a “frequently used acupoint pattern” was identified using descriptive frequency analysis based on cumulative selection frequency. Specifically, acupoints and meridians with the highest usage frequencies and percentages were calculated. The top-ranked high-frequency acupoints (specifically GV20, EX-HN1, and ST36) represent a candidate reference acupoint combination that reflects common clinical practice and TCM compatibility principles. This pattern should be interpreted as hypothesis-generating for future prospective research rather than as an established or superior prescription.

#### 2.4.5 Assessment of primary study overlap

To evaluate the risk of primary study overlap among the included systematic reviews, we calculated the Corrected Covered Area (CCA). The CCA is a widely accepted metric for quantifying the degree of redundancy in overviews, calculated as CCA = (N −r)/[r × (m −1)], where N is the total number of primary study occurrences across all systematic reviews (including double-counting), r is the number of unique primary studies after deduplication, and m is the number of included systematic reviews. In this formula, N represents the total number of primary studies across all reviews including double-counting, r represents the number of unique primary studies after deduplication, and m represents the number of included systematic reviews. According to the thresholds proposed by Pieper et al. (2014), a CCA value between 0% and 5% indicates slight overlap, 6%–10% indicates moderate overlap, 11%–15% indicates high overlap, and greater than 15% indicates very high overlap.

## 3 Results

### 3.1 Literature search and screening results

A total of 277 related literatures were initially retrieved. After removing 91 duplicate literatures, 186 literatures remained. By screening the titles and abstracts, 169 articles were excluded for reasons such as being unrelated studies, literature reviews, protocols, network meta-analyses, or animal experiments. Subsequently, the full texts of the remaining 17 literatures were reviewed, and 1 conference literature was excluded. Ultimately, 16 literatures (Chen et al., 2022; Chen, 2016; Lan et al., 2020; Li et al., 2019; Liao J. et al., 2025; Peng et al., 2004; Song, 2024; Su et al., 2021; Wan et al., 2017; Xie et al., 2014; Xue et al., 2024; Yan et al., 2024; Yuan, 2018; Zhang W. et al., 2023; Zhou et al., 2023; Zhu and Zhang, 2009) were included in this review. The specific screening process is shown in Figure 1. Additionally, the assessment of primary study overlap revealed that the 16 included systematic reviews (m equals 16) contained a total of 318 primary-study occurrences (N equals 318), obtained by summing the per-review trial counts reported in Table 1. After strict deduplication, 242 unique primary RCTs were identified (r equals 242). The calculated CCA value was 2.09%. According to the criteria established by Pieper et al. (2014), this indicates slight overlap among the primary studies, which reduces, but does not eliminate, concerns regarding redundancy and double-counting of primary trial data. The full study-by-review citation matrix is provided in Supplementary File 2.

**FIGURE 1:**
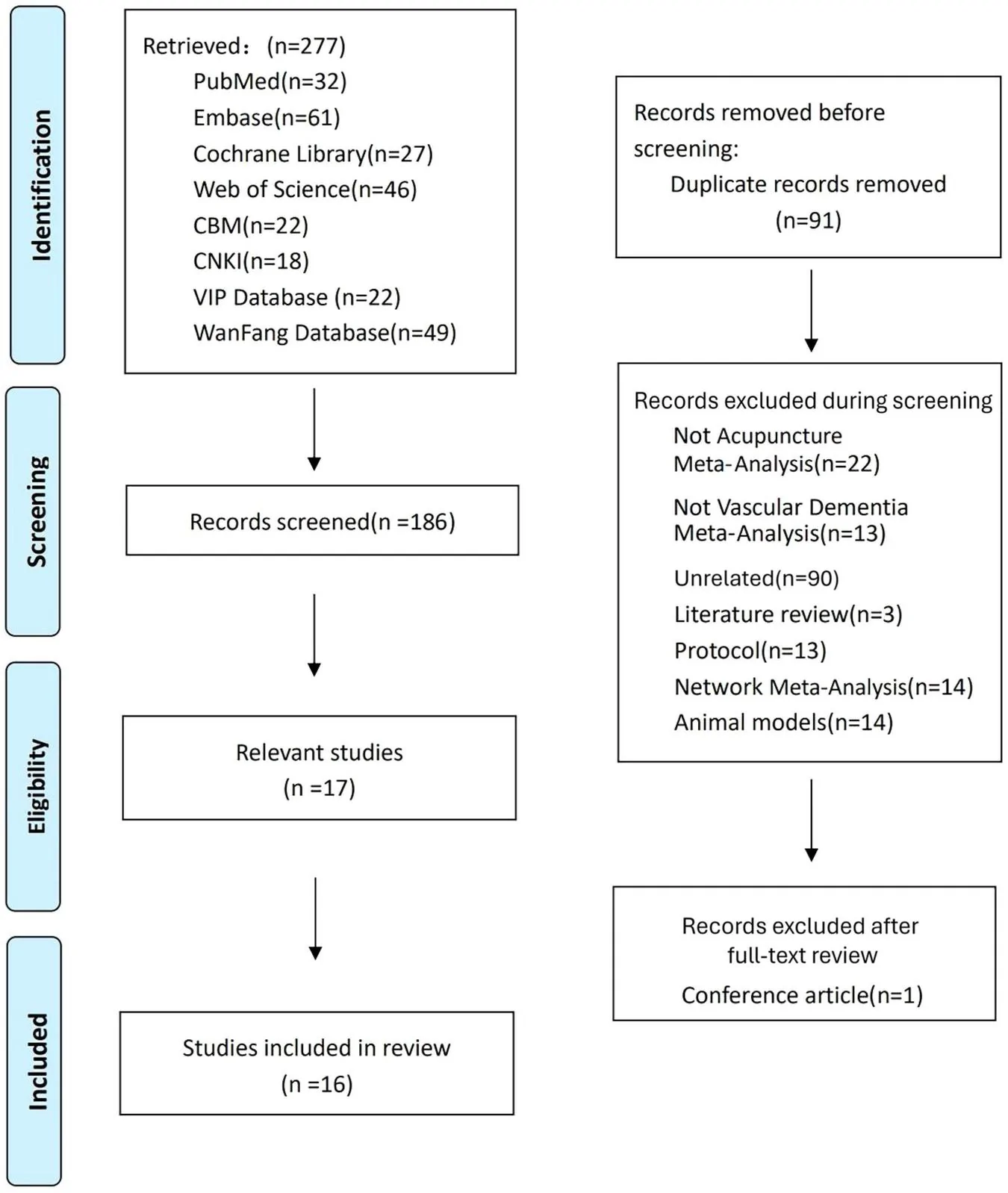
PRISMA flow diagram.

**TABLE 1:** Basic information of the included SRs/MAs.

References	Trials (subjects)	Intervention group	Control group	Quality assessment	Outcome indicators	Main results
Jia et al., 2020	11 (1101)	Acupuncture/ Acupuncture+Routine treatment in Western medicine	Routine treatment in Western medicine	Cochrane	➀, ➁	Acupuncture alone or combined with western medication may be effective in improving cognitive function in patients with vascular dementia.
Su et al., 2021	48 (3778)	Acupuncture/ Acupuncture+Routine treatment in Western medicine/ Acupuncture+Usual Care	Routine treatment in Western medicine/ Usual Care	Cochrane	➀, ➁, ➂, ➃	As a monotherapy or adjunctive therapy, acupuncture may exert positive effects in enhancing cognitive and daily living activities in patients with vascular cognitive impairment, with few adverse effects.
Chen et al., 2022	34 (2672)	Acupuncture	Usual Care	Jadad	➀, ➁, ➂, ➄, ➅, ➆	Acupuncture may be beneficial for vascular dementia, although robust supporting data are currently lacking.
Xue et al., 2024	18 (1617)	Acupuncture+Routine treatment in Chinese herbal medicine	Routine treatment in Western medicine	Cochrane	➀, ➁, ➂, ➇	Acupuncture combined with Chinese herbal medicine demonstrates considerable clinical efficacy in treating vascular dementia, superior to routine treatment in western medication.
Zhu and Zhang, 2009	14 (N/ A)	Electroacupuncture/ Acupuncture+Medicine	Routine treatment in Western medicine/ Acupuncture	Jadad	➇	Electroacupuncture satisfactory outcomes in improving the total effective rate in vascular dementia patients.
Yan et al., 2024	9 (638)	Electroacupuncture+Routine treatment in Western medicine	Routine treatment in Western medicine	Cochrane	➀, ➂, ➆, ➈	Compared with routine treatment in western medication, electroacupuncture combined with Chinese herbal medicine more effectively enhances the total effective rate in vascular dementia patients and better improves their cognitive function, daily living activities, and behavioral performance.
Wan et al., 2017	6 (435)	Electroacupuncture	Routine treatment in Western medicine	Jadad	➀, ➆, ➉	Electroacupuncture at scalp acupoints is more effective than western medication in improving the total effective rate and cognitive function (as measured by MMSE scores) in vascular dementia patients; however, no significant difference was observed between the two therapies in improving daily living activities (as assessed by ADL-R scores).
Peng et al., 2004	5 (308)	Electroacupuncture	Routine treatment in Western medicine	Juni	➁, ➃, ⑪, ⑫	Electroacupuncture is safe for vascular dementia and more effective than the control group in improving global function and cognitive function, with particularly notable efficacy in global function improvement.
Chen, 2016	23 (1706)	Acupuncture/ Acupuncture+Routine treatment in Western medicine	Routine treatment in Western medicine	Cochrane	➀, ➁, ➆	Acupuncture demonstrates advantages in most indicators of cognitive function improvement in vascular dementia patients.
Liao J. et al., 2025	27 (2086)	Acupuncture+Routine treatment in Western medicine	Routine treatment in Western medicine	Cochrane	➀, ➂, ➆	Acupuncture combined with western medication is superior to western medication alone in enhancing the overall response rate, daily living ability (ADL), and mental status (MMSE) in vascular dementia patients.
Zhang X. et al., 2023	10 (798)	Acupuncture+Routine treatment in Chinese herbal medicine	Routine treatment in Western medicine	Cochrane	➀, ➂, ➆	Acupuncture combined with Chinese herbal medicine outperforms western medication alone in improving the overall response rate, mental and cognitive status (MMSE), and quality of life (ADL) in vascular dementia.
Yuan, 2018	15 (1144)	Special acupuncture/ Acupuncture/ Acupuncture+Routine treatment in Western medicine/ Scalp acupuncture	Acupuncture/ Routine treatment in Western medicine/ Rehabilitation	Cochrane	➀, ➁, ➂, ➃, ➄, ➆, ➈	Acupuncture is effective in treating vascular dementia, particularly in MMSE scores, demonstrating positive therapeutic effects.
Li et al., 2019	17 (1283)	Acupuncture	Routine treatment in Western medicine	Cochrane	➀, ➁, ➂, ➆	Acupuncture alone is effective in treating vascular dementia, especially in improving cognitive function, and is superior to routine treatment in medication.
Xie et al., 2014	17 (1074)	Acupuncture+Routine treatment in Chinese herbal medicine	Other treatments	Jadad	➆, ⑬	The combination of acupuncture and Chinese herbal medicine is more effective than other monotherapies in treating vascular dementia, representing an effective and feasible approach.
Song, 2024	51 (4360)	Acupuncture/ Acupuncture+Routine treatment in Western medicine	Routine treatment in Western medicine	Cochrane	➀, ➂, ➆, ⑭, ⑮, ⑯, ⑰	Compared with western medication alone, acupuncture shows better efficacy in treating vascular dementia, with significant advantages in improving cognitive function, clinical symptoms, and reducing serum levels of Hcy and CRP.
Zhang W. et al., 2023	13 (1064)	Acupuncture+Routine treatment in Chinese herbal medicine/ Acupuncture+Routine treatment in Western medicine	Routine treatment in Western medicine/ Routine treatment in Chinese herbal medicine	Cochrane	➀, ➂, ➆, ⑱	The integrated acupuncture and medication regimen is significantly superior to medication alone in improving clinical response rates, cognitive function (MMSE), daily living activities (ADL), and TCM syndrome manifestations.

➀Mini-Mental State Examination (MMSE); ➁Hierarchic Dementia Scale (HDS); ➂Activities of Daily Living Scale (ADL Scale); ➃Adverse events; ➄Scale for the differentiation of syndromes of vascular dementia (SDSVD); ➅NIH stroke scale (NIHSS); ➆Total Effective Rate; ➇Clinical Efficacy; ➈ Blessed behavior scale (BBS); ➉ Activities of Daily Living - Revised (ADL-R); ⑪ Functional Activities Questionnaire (FAQ); ⑫ Global Function; ⑬ Markedly Effective Rate; ⑭ MoCA; ⑮ Barthel ADL Index (BI); ⑯ Hcy level; ⑰ CRP level; ⑱ TCM syndrome manifestation.

### 3.2 The basic characteristics of the included literature

Of the 16 included SRs/MAs, 4 of which were published in English and 12 in Chinese, all studies were conducted in China. The number of RCTs in the included SRs/MAs ranged from 5 to 51, with a sample size of between 308 and 4360. For the quality assessment of the primary studies, 10 SRs/MAs utilized the Cochrane risk-of-bias tool, 5 used the Jadad scale, and 1 employed the Juni quality criteria. The detailed characteristics of the included literature are presented in Table 1.

### 3.3 Results on SRs/MAs quality assessment

#### 3.3.1 Results of the methodological quality

The methodological quality of the 16 SRs/MAs was assessed using the AMSTAR-2 tool. One review was rated as High quality, one as Moderate, six as Low, and eight as Critically Low. The primary factor contributing to the widespread low and critically low ratings was the failure to establish a protocol prior to the review’s commencement (Item 2). This critical flaw was present in 14 of the 16 reviews (87.5%). Other recurring weaknesses included the failure to provide a list of excluded studies with justifications (Item 7), which was a deficit in 5 reviews, and not adequately accounting for the risk of bias from primary studies in the interpretation of the results (Item 13), a flaw noted in 4 reviews. A summary of the AMSTAR-2 assessment is shown in Figure 2 and Table 2.

**FIGURE 2:**
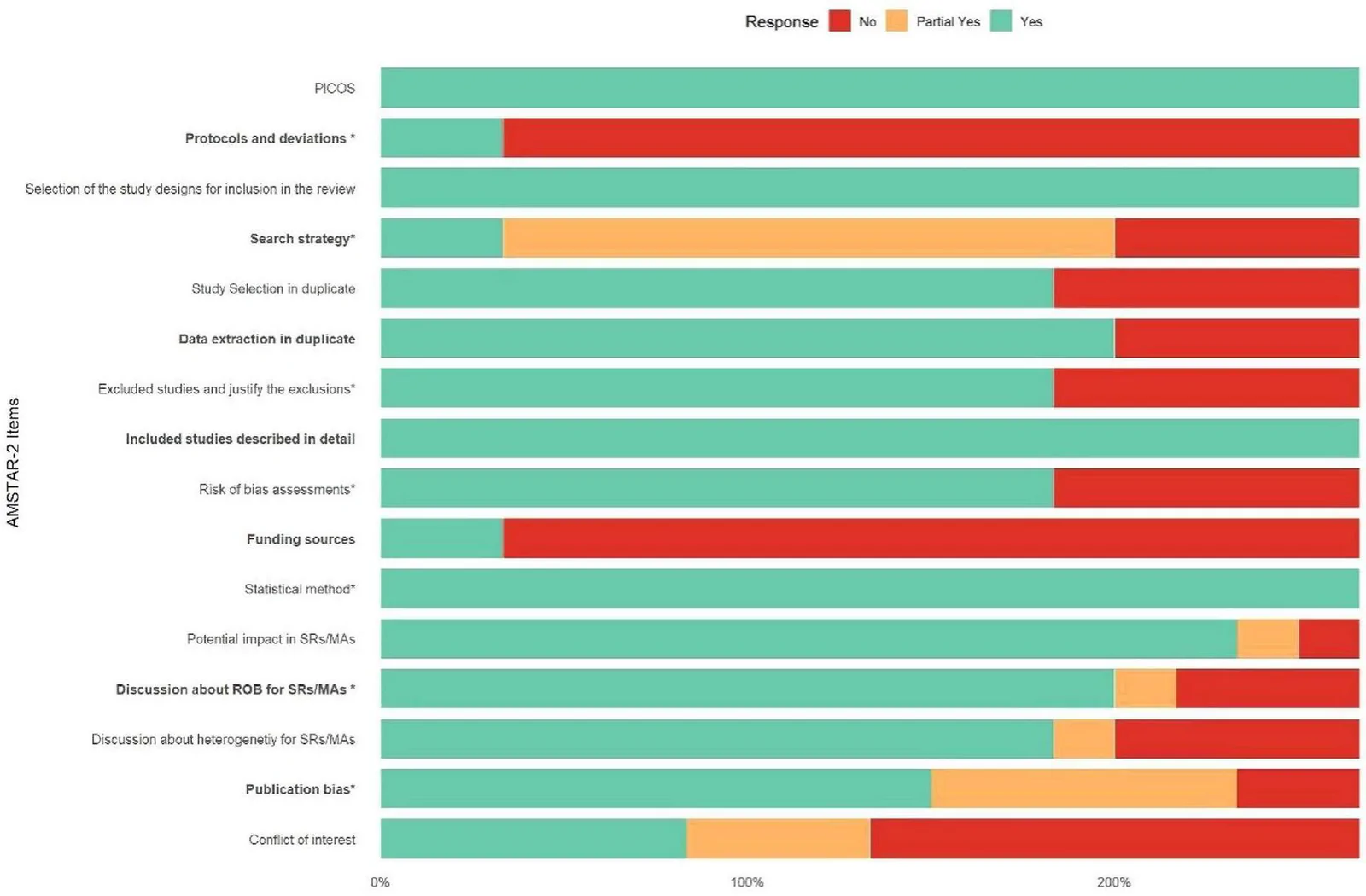
Visualization distribution of the AMSTAR-2 methodological quality. *Critical domains.

**TABLE 2:** Assessment of quality of report by AMASTAR-2 statement.

AMSTAR2	1	2*	3	4*	5	6	7*	8	9*	10	11*	12	13*	14	15*	16	Result
Jia et al., 2020	Y	N	Y	PY	N	Y	Y	Y	Y	N	Y	Y	Y	Y	Y	Y	L
Su et al., 2021	Y	Y	Y	PY	Y	Y	Y	Y	Y	Y	Y	Y	Y	Y	Y	Y	H
Chen et al., 2022	Y	N	Y	PY	Y	Y	Y	Y	N	N	Y	Y	Y	Y	Y	Y	CL
Xue et al., 2024	Y	Y	Y	PY	Y	Y	Y	Y	Y	N	Y	Y	PY	PY	PY	Y	M
Zhu and Zhang, 2009	Y	N	Y	N	N	N	N	N	N	N	Y	Y	N	Y	Y	N	CL
Yan et al., 2024	Y	N	Y	PY	Y	Y	Y	Y	Y	N	Y	Y	Y	Y	Y	PY	L
Wan et al., 2017	Y	N	Y	N	Y	N	N	Y	N	N	Y	Y	Y	N	PY	PY	CL
Peng et al., 2004	Y	N	Y	Y	Y	Y	Y	Y	N	N	Y	N	Y	N	N	N	CL
Chen, 2016	Y	N	Y	Y	Y	Y	N	Y	Y	Y	Y	Y	Y	Y	Y	N	CL
Liao J. et al., 2025	Y	N	Y	N	N	N	N	Y	Y	N	Y	Y	N	N	Y	Y	CL
Zhang X. et al., 2023	Y	N	Y	PY	Y	Y	Y	Y	Y	N	Y	Y	Y	Y	Y	N	L
Yuan, 2018	Y	N	Y	PY	Y	Y	Y	Y	Y	N	Y	Y	Y	N	PY	N	L
Li et al., 2019	Y	N	Y	PY	Y	Y	Y	Y	Y	N	Y	Y	Y	Y	Y	PY	L
Xie et al., 2014	Y	N	Y	PY	N	N	N	Y	N	N	Y	Y	N	Y	Y	N	CL
Song, 2024	Y	N	Y	PY	N	Y	Y	Y	Y	N	Y	Y	Y	Y	Y	N	L
Zhang W. et al., 2023	Y	N	Y	PY	Y	Y	Y	Y	Y	N	Y	Y	Y	Y	Y	N	L

Y = Yes, N = No, PY = Partial Yes, H = High, M = Moderate, L = Low, CL = Critical Low. *Indicate important items.

#### 3.3.2 Completeness of reporting of the included SRs/MAs

The completeness of reporting of the included SRs/MAs was evaluated against the PRISMA 2020 checklist. While many items were well-reported, several key deficiencies were noted. The most significant omission was Item 5 (Protocol and registration), which was adequately reported in only 12.5% (2/16) of the reviews, indicating a widespread lack of prospective registration. Another notable area of incomplete reporting was Item 27 (Funding), with only 56.25% (9/16) of the reviews providing information on their funding sources. Other items with minor reporting gaps included Item 9 (Study selection) and Item 10 (Data collection process), each fully reported in 13 of the 16 reviews (81.25%). In contrast, items such as Item 15 and 22 (Risk of bias across studies) were well-reported, with a completion rate of 87.5%. The detailed results of the PRISMA checklist assessment are available in Figure 3 and Table 3.

**FIGURE 3:**
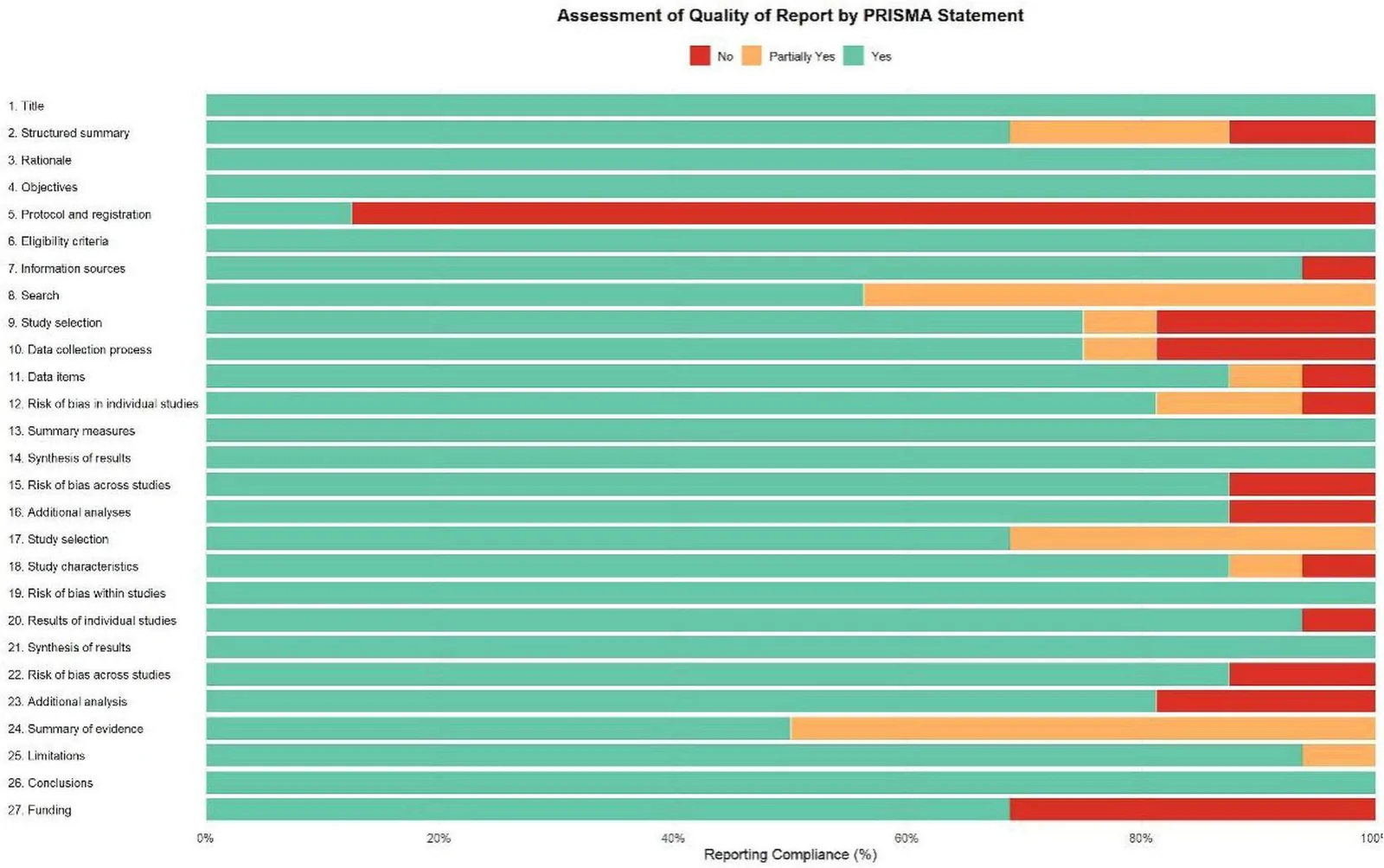
Visualization distribution of the PRISMA methodological.

**TABLE 3:** Assessment of quality of report by PRISMA statement.

Items	Jia et al., 2020	Su et al., 2021	Chen et al., 2022	Xue et al., 2024	Zhu and Zhang, 2009	Yan et al., 2024	Wan et al., 2017	Peng et al., 2004	Chen, 2016	Liao J. et al., 2025	Zhang X. et al., 2023	Yuan, 2018	Li et al., 2019	Xie et al., 2014	Song, 2024	Zhang W. et al., 2023
1. Title	Y	Y	Y	Y	Y	Y	Y	Y	Y	Y	Y	Y	Y	Y	Y	Y
2. Structured summary	PY	Y	Y	Y	PY	Y	Y	N	Y	PY	Y	Y	Y	N	Y	Y
3. Rationale	Y	Y	Y	Y	Y	Y	Y	Y	Y	Y	Y	Y	Y	Y	Y	Y
4. Objectives	Y	Y	Y	Y	Y	Y	Y	Y	Y	Y	Y	Y	Y	Y	Y	Y
5. Protocol and registration	N	Y	N	Y	N	N	N	N	N	N	N	N	N	N	N	N
6. Eligibility criteria	Y	Y	Y	Y	Y	Y	Y	Y	Y	Y	Y	Y	Y	Y	Y	Y
7. Information sources	Y	Y	Y	Y	N	Y	Y	Y	Y	Y	Y	Y	Y	Y	Y	Y
8. Search	Y	Y	Y	Y	PY	Y	Y	PY	Y	PY	PY	Y	PY	PY	Y	PY
9. Study selection	PY	Y	Y	Y	N	Y	Y	Y	Y	N	Y	Y	Y	N	Y	Y
10. Data collection process	Y	Y	Y	Y	N	Y	PY	Y	Y	N	Y	Y	Y	N	Y	Y
11. Data items	Y	Y	Y	Y	N	Y	Y	Y	Y	Y	Y	Y	Y	PY	Y	Y
12. Risk of bias in individual studies	Y	Y	PY	Y	N	Y	PY	Y	Y	Y	Y	Y	Y	Y	Y	Y
13. Summary measures	Y	Y	Y	Y	Y	Y	Y	Y	Y	Y	Y	Y	Y	Y	Y	Y
14. Synthesis of results	Y	Y	Y	Y	Y	Y	Y	Y	Y	Y	Y	Y	Y	Y	Y	Y
15. Risk of bias across studies	Y	Y	Y	Y	Y	Y	N	Y	Y	N	Y	Y	Y	Y	Y	Y
16. Additional analyses	Y	Y	Y	Y	Y	Y	Y	Y	Y	N	Y	Y	Y	N	Y	Y
17. Study selection	Y	Y	Y	Y	PY	Y	PY	PY	Y	PY	Y	Y	Y	PY	Y	Y
18. Study characteristics	Y	Y	Y	Y	PY	Y	Y	Y	Y	Y	Y	Y	Y	N	Y	Y
19. Risk of bias within studies	Y	Y	Y	Y	Y	Y	Y	Y	Y	Y	Y	Y	Y	Y	Y	Y
20. Results of individual studies	Y	Y	Y	Y	Y	Y	Y	N	Y	Y	Y	Y	Y	Y	Y	Y
21. Synthesis of results	Y	Y	Y	Y	Y	Y	Y	Y	Y	Y	Y	Y	Y	Y	Y	Y
22. Risk of bias across studies	Y	Y	Y	Y	Y	Y	N	Y	Y	N	Y	Y	Y	Y	Y	Y
23. Additional analysis	Y	Y	Y	Y	Y	Y	Y	N	Y	N	Y	Y	Y	N	Y	Y
24. Summary of evidence	PY	Y	PY	PY	PY	PY	PY	PY	Y	PY	Y	Y	Y	Y	Y	Y
25. Limitations	Y	Y	Y	Y	PY	Y	Y	Y	Y	Y	Y	Y	Y	Y	Y	Y
26. Conclusions	Y	Y	Y	Y	Y	Y	Y	Y	Y	Y	Y	Y	Y	Y	Y	Y
27. Funding	Y	Y	Y	Y	Y	Y	Y	N	N	Y	Y	N	Y	Y	N	N

Y, yes (a complete report); PY, partially yes (a partially compliant report); N, no (no report).

#### 3.3.3 Results of the quality of the evidence

The GRADE system was used to assess the quality of evidence for 97 individual outcomes across the 16 SRs/MAs. The overall quality of evidence was found to be overwhelmingly low. Of the 97 outcomes, only one (1.0%) was rated as High quality and one (1.0%) as Moderate quality. The vast majority were rated as Low (14 outcomes, 14.4%) or Very Low (81 outcomes, 83.5%). The primary reason for downgrading the evidence was study limitations (risk of bias in the primary RCTs), which affected all 97 outcomes. The next most frequent reason for downgrading was imprecision (wide or very wide confidence intervals), which impacted 57 outcomes (58.8%). This was followed by inconsistency (high heterogeneity), affecting 47 outcomes (48.5%), and suspected publication bias, affecting 36 outcomes (37.1%). The results of the GRADE assessment are detailed in Figure 4 and Table 4.

**FIGURE 4:**
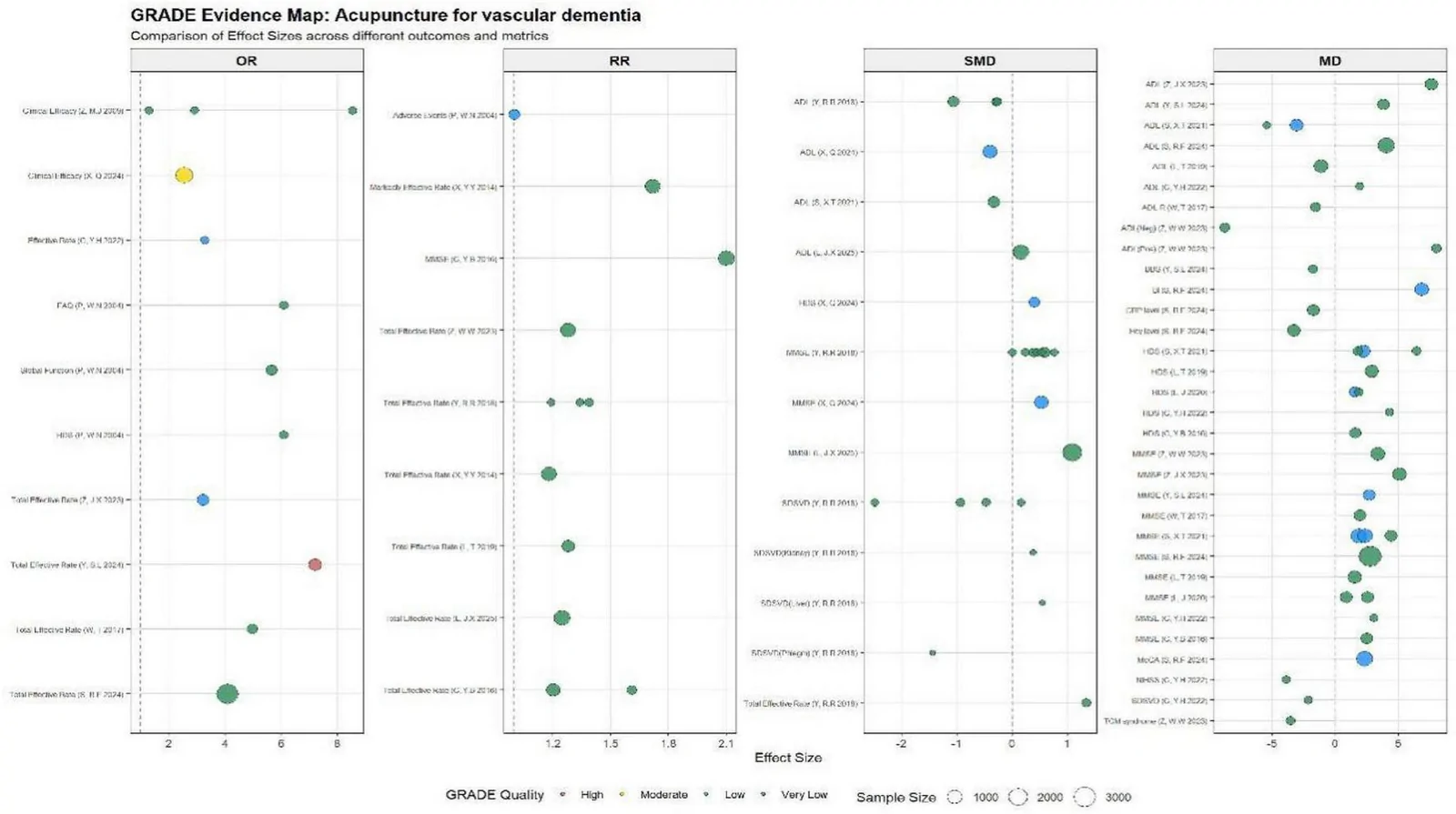
Visualization distribution of the GRADE methodological.

**TABLE 4:** GRADE assessment of included systematic reviews.

References	Outcomes	Intervention group/control group	RCTs/ participants	Limita- tions	Inconsis- tency	Indirect- ness	Impreci- sion	Publication bias	Large effect	Relative effect (95% CI)	Quality
Jia et al., 2020	MMSE	Acupuncture vs. Routine treatment in Western medicine	6/553	−1^a^	−1^c^	0	−1^e^	0	0	MD: 0.87, 95% CI (−0.41, 2.15)	VL
		Acupuncture+Routine treatment in Western medicine vs. Routine treatment in Western medicine	5/512	−1^a^	−2^c^	0	0	0	0	MD: 2.54, 95% CI (0.96, 4.12)	VL
	HDS	Acupuncture vs. Routine treatment in Western medicine	5/413	−1^a^	0	0	−1^e^	−1^g^	0	MD: 1.55, 95% CI (0.72, 2.38),	L
		Acupuncture+Routine treatment in Western medicine vs. Routine treatment in Western medicine	2/131	−1^a^	0	0	−2^e^	0	0	MD: 1.84, 95% CI (0.35, 3.33)	VL
Su et al., 2021	MMSE	Acupuncture vs. Routine treatment in Western medicine	18/1229	−1^a^	−1^c^	0	0	0	0	MD: 1.86, 95% CI (1.19, 2.54)	L
		Acupuncture+Routine treatment in Western medicine vs. Routine treatment in Western medicine	14/1106	−1^a^	−1^c^	0	0	0	0	MD: 2.37, 95% CI (1.60, 3.14)	L
		Acupuncture+Usual care vs. Usual care	7/530	−1^a^	−1^c^	0	1^e^	−1^g^	0	MD: 4.40, 95% CI (1.61, 7.19)	VL
	HDS	Acupuncture vs. Routine treatment in Western medicine	12/781	−1^a^	−1^c^	0	0	0	0	MD: 2.20, 95% CI (1.27, 3.1)	L
		Acupuncture+Routine treatment in Western medicine vs. Routine treatment in Western medicine	4/235	−1^a^	0	0	−1^e^	−1^g^	0	MD: 1.77, 95% CI (0.85, 2.69)	VL
		Acupuncture+Usual care vs. Usual care	2/177	−1^a^	0	0	−1^e^	−1^g^	0	MD 6.42, 95% CI (5.04, 7.81)	VL
	ADL Scale	Acupuncture vs. Routine treatment in Western medicine	7/704	−1^a^	−1^c^	0	0	0	0	MD: −3.08, 95% CI (−4.81, −1.35)	L
		Acupuncture+Routine treatment in Western medicine vs. Routine treatment in Western medicine	5/466	−1^a^	0	0	−1^e^	−1^g^	0	SMD: −0.34, 95% CI (−0.52, −0.16)	VL
		Acupuncture+Usual Care vs. Usual Care	1/70	−1^a^	0	0	−1^e^	−1^g^	0	MD: −5.43, 95% CI (−7.27, −3.59)	VL
	AEs	Acupuncture vs. Routine treatment in Western medicine	3/461	−1^a^	0	0	−1^f^	−1^g^	0	RR: 0.80, 95% CI (0.35, 1.81)	VL
		Acupuncture+Routine treatment in Western medicine vs. Routine treatment in Western medicine	3/262	−1^a^	0	0	−1^f^	−1^g^	0	RR: 0.76, 95% CI (0.29, 2.05)	VL
		Acupuncture+Usual care vs. Usual care	1/77	−1^a^	0	0	−1^f^	−1^g^	0	RR: 0.51, 95% CI (0.05, 5.43)	VL
Chen et al., 2022	Effective Rate	Acupuncture vs. Usual care	26/(N/A)	−1^a^	0	−1^d^	0	0	0	OR: 3.28, 95% CI (2.54, 4.24)	L
	HDS	Acupuncture vs. Usual care	12/(N/A)	−1^a^	−1^c^	−1^d^	0	0	0	WMD: 4.31, 95% CI (3.15, 5.47)	VL
	MMSE	Acupuncture vs. Usual care	29/(N/A)	−1^a^	−1^c^	−1^d^	0	−1^g^	0	WMD: 3.07, 95% CI (2.40, 3.74)	VL
	ADL	Acupuncture vs. Usual care	24/(N/A)	−1^a^	−2^c^	−1^d^	−1^f^	0	0	WMD: 1.93, 95% CI (−2.53, 6.38)	VL
	SDSVD	Acupuncture vs. Usual care	5/(N/A)	−1^a^	−1^c^	−1^d^	−1^e^	−1^g^	0	WMD: −2.15, 95% CI (−4.14, −0.16)	VL
	NIHSS	Acupuncture vs. Usual care	5/(N/A)	−1^a^	0	−1^d^	−1^e^	−1^g^	0	WMD: −3.90, 95% CI (−4.87, −2.94)	VL
Xue et al., 2024	Clinical Efficacy	Acupuncture+Routine treatment in Chinese herbal medicine vs. Routine treatment in Western medicine	17/1547	−1^a^	0	0	0	−1^g^	+1	OR: 2.54, 95% CI (1.98, 3.26)	M
	MMSE	Acupuncture+Routine treatment in Chinese herbal medicine vs. Routine treatment in Western medicine	12/807	−1^a^	−1^c^	0	0	0	0	SMD: 0.52, 95% CI (0.25, 0.78)	L
	ADL	Acupuncture+Routine treatment in Chinese herbal medicine vs. Routine treatment in Western medicine	10/903	−1^a^	−1^c^	0	0	0	0	SMD: −0.40, 95% CI (−0.69, −0.11)	L
	HDS	Acupuncture+Routine treatment in Chinese herbal medicine vs. Routine treatment in Western medicine	5/327	−1^a^	0	0	−1^e^	0	0	SMD: 0.40, 95% CI (0.18, 0.62)	L
Zhu and Zhang, 2009	Clinical Efficacy	Electroacupuncturevs. Routine treatment in Western medicine	7/(N/A)	−2^b^	−1^c^	0	0	−1^g^	0	OR: 2.91, 95% CI (1.86, 4.64)	VL
		Acupuncture+Medicine vs. Acupuncture	2/(N/A)	−2^b^	−1^c^	0	−1^f^	0	0	OR: 1.29, 95% CI (0.23, 7.38)	VL
		Acupuncture+Medicine vs. Routine treatment in Western medicine	3/(N/A)	−2^b^	−1^c^	0	−1^f^	0	0	OR: 8.53, 95% CI (4.30, 16.88)	VL
Yan et al., 2024	Total Effective Rate	Electroacupuncture+Routine treatment in Western medicine vs. Routine treatment in Western medicine	8/574	−1^a^	0	0	0	0	+1	OR: 7.19, 95% CI (4.61, 11.21)	H
	MMSE	Electroacupuncture+Routine treatment in Western medicine vs. Routine treatment in Western medicine	7/474	−1^a^	−1^c^	0	0	0	0	MD: 2.65, 95% CI (1.47, 3.82)	L
	ADL	Electroacupuncture+Routine treatment in Western medicine vs. Routine treatment in Western medicine	7/474	−1^a^	−1^c^	0	−1^f^	0	0	MD: 3.78, 95% CI (0.32, 7.25)	VL
	BBS	Electroacupuncture+Routine treatment in Western medicine vs. Routine treatment in Western medicine	2/124	−1^a^	0	0	−1^e^	−1^g^	0	MD: −1.79, 95% CI (−2.28, −1.29)	VL
Wan et al., 2017	MMSE	Electroacupuncture vs. Routine treatment in Western medicine	6/435	−2^b^	−1^c^	0	−1^e^	−1^g^	0	MD: 1.92, 95% CI (0.82, 3.03)	VL
	ADL-R	Electroacupuncture vs. Routine treatment in Western medicine	3/264	−2^b^	0	0	−1^f^	−1^g^	0	MD: −1.55, 95% CI (−4.65, 1.54)	VL
	Total Effective Rate	Electroacupuncture vs. Routine treatment in Western medicine	3/264	−2^b^	−1^c^	−1^d^	0	−1^g^	0	OR: 4.97, 95% CI (2.89, 8.56)	VL
Peng et al., 2004	Global Function	Electroacupuncture vs. Routine treatment in Western medicine	5/308	−1^a^	0	0	−1^e^	−1^g^	0	OR: 5.64, 95% CI (2.87, 11.09)	VL
	HDS	Electroacupuncture vs. Routine treatment in Western medicine	2/134	−1^a^	0	0	−2^f^	−1^g^	0	OR: 6.07, 95% CI (3.76, 8.38)	VL
	FAQ	Electroacupuncture vs. Routine treatment in Western medicine	2/134	−1^a^	0	0	−2^f^	−1^g^	0	OR = 6.07, 95% CI (−3.21, 0.74)	VL
	Adverse Events	Electroacupuncture vs. Routine treatment in Western medicine	5/308	−1^a^	0	0	−1^e^	0	0	No adverse events were reported.	L
Chen, 2016	Total Effective Rate	Acupuncture vs. Routine treatment in Western medicine	9/751	−2^b^	0	0	−1^e^	−1^g^	0	RR = 1.20, 95% CI (1.03, 1.40)	VL
		Acupuncture+Routine treatment in Western medicine vs. Routine treatment in Western medicine	2/233	−1^a^	−2^c^	0	−1^e^	0	0	RR = 1.61, 95% CI (1.02, 2.53)	VL
	MMSE	Acupuncture vs. Routine treatment in Western medicine	17/1255	−2^b^	−1^c^	0	0	−1^g^	0	RR = 2.10, 95% CI (1.37, 2.83)	VL
		Acupuncture+Routine treatment in Western medicine vs. Routine treatment in Western medicine	5/436	−2^b^	0	0	0	−1^g^	0	MD = 2.46, 95% CI (1.69, 3.23)	VL
	HDS	Acupuncture vs. Routine treatment in Western medicine	5/396	−2^b^	0	0	−1^e^	0	0	MD = 1.59, 95% CI (0.75, 2.44)	VL
Liao J. et al., 2025	Total Effective Rate	Acupuncture+Routine treatment in Western medicine vs. Routine treatment in Western medicine	17/1313	−2^b^	0	0	0	−1^g^	0	RR = 1.25, 95% CI (1.18, 1.32)	VL
	ADL	Acupuncture+Routine treatment in Western medicine vs. Routine treatment in Western medicine	17/1298	−2^b^	−1^c^	0	0	−1^g^	0	SMD = 0.16, 95% CI (0.04, 0.28)	VL
	MMSE	Acupuncture+Routine treatment in Western medicine vs. Routine treatment in Western medicine	25/2098	−2^b^	−1^c^	0	0	−1^g^	0	SMD = 1.09, 95% CI (0.73, 1.46)	VL
Zhang X. et al., 2023	Total Effective Rate	Acupuncture+Routine treatment in Chinese herbal medicine vs. Routine treatment in Western medicine	6/442	−1^a^	0	0	−1^e^	0	0	OR = 3.21, 95% CI (1.90, 5.42)	L
	MMSE	Acupuncture+Routine treatment in Chinese herbal medicine vs. Routine treatment in Western medicine	10/798	−1^a^	−2^c^	0	−1^f^	0	0	MD = 5.06, 95% CI (1.10, 9.02)	VL
	ADL	Acupuncture+Routine treatment in Chinese herbal medicine vs. Routine treatment in Western medicine	7/584	−1^a^	−1^c^	0	−1^e^	0	0	MD = 7.59, 95% CI (5.66, 9.52)	VL
Yuan, 2018	Total Effective Rate	Special acupuncture vs. Acupuncture	2/135	−2^b^	−1^c^	0	−1^e^	0	0	RR = 1.39, 95% CI (0.89, 2.16)	VL
		Acupuncture vs. Almitrine and Raubasine	2/120	−2^b^	−1^e^	0	0	0	0	SMD = 1.34, 95% CI (1.04, 1.73)	VL
		Acupuncture+Almitrine and Raubasine vs. Almitrine and Raubasine	1/65	−2^b^	0	0	−2^f^	0	0	RR = 1.34, 95% CI (1.01, 1.77)	VL
		Acupuncture+Oxiracetam vs. Oxiracetam	1/70	−2^b^	0	0	−2^f^	0	0	RR = 1.19, 95% CI (0.96, 1.46)	VL
	MMSE	Acupuncture+Routine treatment in Western medicine vs. Acupuncture	4/216	−2^b^	0	−1^d^	−1^e^	0	0	SMD = 0.39, 95% CI (0.13, 0.64)	VL
		Acupuncture vs. Donepezil	2/100	−2^b^	−1^c^	0	−1^e^	0	0	SMD = 0.24, 95% CI (−0.37 = 0.85)	VL
		Acupuncture vs. Almitrine and Raubasine	2/120	−2^b^	−1^e^	0	0	0	0	SMD = 0.44, 95% CI (0.07, 0.80)	VL
		Acupuncture vs. Nimodipine	3/377	−2^b^	−2^c^	0	0	0	0	SMD = 0.58, 95% CI (−0.04, 1.20)	VL
		Acupuncture vs. Ergoloid Mesylates	1/60	−2^b^	0	0	−2^f^	0	0	SMD = 0.76, 95% CI (0.23, 1.28)	VL
		Acupuncture+Almitrine and Raubasine vs. Almitrine and Raubasine	1/65	−2^b^	0	0	−2^f^	0	0	SMD = 0.53, 95% CI (0.03, 1.02)	VL
		Acupuncture+Oxiracetam vs. Oxiracetam	1/70	−2^b^	0	0	−2^f^	0	0	SMD = 0, 95% CI (−0.47, 0.47)	VL
	SDSVD	Special acupuncture vs. Acupuncture	2/126	−2^b^	−2^c^	0	−1^f^	0	0	SMD = −0.94, 95% CI (−2.10, 0.22)	VL
		Acupuncture vs. Donepezil	2/100	−2^b^	−1^e^	0	0	0	0	SMD = −0.47, 95% CI (−0.87, −0.07)	VL
		Acupuncture vs. Ergoloid Mesylates	1/60	−2^b^	0	0	−2^f^	0	0	SMD = −2.49, 95% CI (−3.18, −1.81)	VL
	SDSVD (pattern of kidney deficiency and essence depletion)	Acupuncture vs. Rehabilitation	1/22	−2^b^	0	0	−2^f^	0	0	SMD = 0.38, 95% CI (−0.48, 1.24)	VL
	SDSVD (pattern of phlegm-turbidity obstruction)	Acupuncture vs. Rehabilitation	1/13	−2^b^	0	0	−2^f^	0	0	SMD = −1.44, 95% CI (−2.79, −0.09)	VL
	SDSVD (pattern of Liver Yang Hyperactivity)	Acupuncture vs. Rehabilitation	1/11	−2^b^	0	0	−2^f^	0	0	SMD = 0.54, 95% CI (−0.82, 1.89)	VL
		Acupuncture vs. Rehabilitation	1/63	−2^b^	0	0	−2^f^	0	0	SMD = 0.16, 95% CI (−0.30, 0.63)	VL
	ADL	Special acupuncture vs. Acupuncture	2/121	−2^b^	0	0	−1^e^	0	0	SMD = −0.29, 95% CI (−0.68, 0.18)	VL
		Acupuncture vs. Donepezil	2/100	−2^b^	−1^c^	0	−1^e^	0	0	SMD = −0.27, 95% CI (−0.89, 0.36)	VL
		Acupuncture vs. Nimodipine	3/377	−2^b^	−2^c^	0	0	0	0	SMD = −1.06, 95% CI (−3.08, 0.96)	VL
		Acupuncture+Almitrine and Raubasinevs. Almitrine and Raubasine	1/65	−2^b^	0	0	−2^f^	0	0	SMD = −0.62, 95% CI (−1.12, −0.12)	VL
		Acupuncture+Oxiracetam vs. Oxiracetam	1/70	−2^b^	0	0	−2^f^	0	0	SMD = −0.13, 95% CI (−0.60, 0.34)	VL
	HDS	Acupuncture vs. Almitrine and Raubasine	2/120	−2^b^	−1^c^	0	0	0	0	SMD = 0.25, 95% CI (−0.27, 0.77)	VL
		Acupuncture+Almitrine and Raubasinevs. Almitrine and Raubasine	1/65	−2^b^	0	0	−2^f^	0	0	SMD = −2.93, 95% CI (−3.64, −2.22)	VL
		Acupuncture+Oxiracetam vs. Oxiracetam	1/70	−2^b^	0	0	−2^f^	0	0	SMD = 0.16, 95% CI (−0.30, 0.63)	VL
	BBS	Acupuncture vs. Ergoloid Mesylates	1/60	−2^b^	0	0	−2^f^	0	0	SMD = −0.76, 95% CI (−1.28, −0.23)	VL
	Adverse Events	Scalp acupuncture vs. Acupuncture	1/60	−2^b^	0	0	−2^f^	0	0	RR = 3.0, 95% CI (0.13, 70.83)	VL
Li et al., 2019	Total Effective Rate	Acupuncture vs. Routine treatment in Western medicine	10/614	−2^b^	−1^c^	−1^d^	0	−1^g^	0	RR = 1.28, 95% CI (1.14, 1.44)	VL
	HDS	Acupuncture vs. Routine treatment in Western medicine	9/716	−2^b^	0	−1^d^	0	0	0	MD = 2.89, 95% CI (1.89, 3.89)	VL
	MMSE	Acupuncture vs. Routine treatment in Western medicine	9/747	−2^b^	−1^c^	−1^d^	0	0	0	MD = 1.49, 95% CI (0.91, 2.07)	VL
	ADL	Acupuncture vs. Routine treatment in Western medicine	11/916	−2^b^	−2^c^	0	−1f	0	0	MD = −1.12, 95% CI (−6.91, 4.67)	VL
Xie et al., 2014	Total Effective Rate	Acupuncture+Routine treatment in Chinese herbal medicine vs. Other treatments	17/1074	−2^b^	−1^c^	−1^d^	0	−1^g^	0	RR = 1.18, 95% CI (1.12, 1.25)	VL
	Markedly Effective Rate	Acupuncture+Routine treatment in Chinese herbal medicine vs. Other treatments	16/1036	−2^b^	−1^c^	−1^d^	0	−1^g^	0	RR = 1.72, 95% CI (1.44, 2.05)	VL
Song, 2024	Total Effective Rate	Acupuncture/Acupuncture+Routine treatment in Western medicine vs. Routine treatment in Western medicine	35/3075	−1^a^	0	−1^d^	0	−1^g^	0	OR = 4.07, 95% CI (3.34, 4.96)	VL
	MMSE	Acupuncture/Acupuncture+Routine treatment in Western medicine vs. Routine treatment in Western medicine	42/3299	−1^a^	−1^c^	0	0	−1^g^	0	WMD = 2.74, 95% CI (2.17, 3.31)	VL
	MoCA	Acupuncture/Acupuncture+Routine treatment in Western medicine vs. Routine treatment in Western medicine	15/1313	−1^a^	−1^c^	0	0	0	0	MD = 2.32, 95% CI (1.87, 2.77)	L
	ADL	Acupuncture/Acupuncture+Routine treatment in Western medicine vs. Routine treatment in Western medicine	19/1394	−1^a^	−1^c^	0	−1^f^	−1^g^	0	MD = 4.03, 95% CI (0.70, 7.37)	VL
	BI	Acupuncture/Acupuncture+Routine treatment in Western medicine vs. Routine treatment in Western medicine	8/776	−1^a^	−1^c^	0	0	0	0	MD = 6.81, 95% CI (3.93, 9.70)	L
	Hcy level	Acupuncture/Acupuncture+Routine treatment in Western medicine vs. Routine treatment in Western medicine	7/629	−1^a^	−2^c^	−1^d^	0	0	0	MD = −3.31, 95% CI (−5.63, −0.98)	VL
	CRP level	Acupuncture/Acupuncture+Routine treatment in Western medicine vs. Routine treatment in Western medicine	5/426	−1^a^	−1^c^	−1^d^	0	0	0	MD = −1.76, 95% CI (−2.45, −1.06)	VL
Zhang W. et al., 2023	Total Effective Rate	Acupuncture+Routine treatment in Chinese herbal medicine/Acupuncture+Routine treatment in Western medicine vs. Routine treatment in Western medicine/Routine treatment in Chinese herbal medicine	13/1064	−2^b^	0	0	0	−1^g^	0	RR = 1.28, 95% CI (1.20, 1.37)	VL
	MMSE	Acupuncture+Routine treatment in Chinese herbal medicine/Acupuncture+Routine treatment in Western medicine vs. Routine treatment in Western medicine/Routine treatment in Chinese herbal medicine	11/870	−2^b^	−1^c^	0	0	−1^g^	0	MD = 3.35, 95% CI (2.18, 4.51)	VL
	ADL (Positively oriented scores)	Acupuncture+Routine treatment in Chinese herbal medicine/Acupuncture+Routine treatment in Western medicine vs. Routine treatment in Western medicine/Routine treatment in Chinese herbal medicine	3/228	−2^b^	0	0	−1^e^	−1^g^	0	MD = 7.99, 95% CI (6.14, 9.83)	VL
	ADL (Negatively oriented scores)	Acupuncture+Routine treatment in Chinese herbal medicine/Acupuncture+Routine treatment in Western medicine vs. Routine treatment in Western medicine/Routine treatment in Chinese herbal medicine	3/236	−2^b^	−1^c^	0	−1^e^	−1^g^	0	MD = −8.80, 95% CI (−12.40, −5.19)	VL
	TCM syndrome manifestations	Acupuncture+Routine treatment in Chinese herbal medicine/Acupuncture+Routine treatment in Western medicine vs. Routine treatment in Western medicine/Routine treatment in Chinese herbal medicine	2/190	−2^b^	−1^c^	0	−1^e^	−1^g^	0	MD = −3.54, 95% CI (−4.46, −2.63)	VL

^a^Inadequate allocation concealment;

^b^Inadequate allocation concealment and lack of blinding;

^c^High heterogeneity;

^d^Indirect evidence;

^e^Wide confidence interval;

^f^Very wide confidence interval;

^g^Funnel plot asymmetry or other factors indicating publication bias. H, High; M, Moderate; L, Low; VL, Very Low.

#### 3.3.4 Summary of results

##### 3.3.4.1 Clinical effectiveness

A total of 14 SRs/MAs reported on clinical effectiveness, using metrics such as Total Effective Rate, Clinical Efficacy, or Global Function. The majority of reviews concluded that acupuncture, whether as a monotherapy or combined with medication, demonstrated superior therapeutic efficacy compared to medication alone. Notably, the review by Yan et al. (2024) provided High-quality evidence that electroacupuncture combined with routine Western medicine was significantly more effective than Western medicine alone (OR = 7.19, 95% CI [4.61, 11.21]). Furthermore, the review by Xue et al. (2024) offered Moderate-quality evidence that the combined application of acupuncture and Chinese herbal medicine showed superior therapeutic efficacy compared to routine Western medicine (OR = 2.54, 95% CI [1.98, 3.26]). The evidence from the other 12 reviews, while supporting the positive effect of acupuncture, was rated as Low or Very Low.

##### 3.3.4.2 Cognitive function

A total of 14 SRs/MAs reported on cognitive function, primarily using the MMSE and HDS. The review by Su et al. (2021) conducted several subgroup analyses, yielding the following results: (1) Acupuncture as a standalone treatment was superior to routine Western medicine in improving both MMSE and HDS scores (Low-quality evidence); (2) The combined application of acupuncture and routine Western medicine demonstrated superior efficacy in improving MMSE and HDS scores compared to Western medicine alone (Low and Very Low-quality evidence, respectively).

Other reviews consistently reported that both acupuncture monotherapy and combined therapy were more effective in improving cognitive scores than control interventions. However, the evidence quality for these outcomes was predominantly rated as Low or Very Low due to study limitations, inconsistency, and/or imprecision.

##### 3.3.4.3 Activities of daily living

A total of 12 SRs/MAs reported on activities of daily living, using scales such as the ADL Scale, BI, FAQ, or ADL-R. The review by Su et al. (2021) reported that acupuncture alone or combined with usual care was more effective than usual care alone in improving ADL scores (Low and Very Low-quality evidence, respectively). Furthermore, 11 other SRs/MAs reported that the combined application of acupuncture with Western or Chinese medicine showed superior therapeutic efficacy in improving ADL compared to medication alone. However, the evidence supporting these findings was consistently rated as Low or Very Low due to factors such as study limitations, high inconsistency, and imprecision.

##### 3.3.4.4 Safety

Three SRs/MAs (Peng et al., 2004; Su et al., 2021; Yuan, 2018) explicitly included adverse events as an outcome. Available data did not identify a clear increase in reported adverse events; however, the certainty of the safety evidence is very low and adverse-event ascertainment in the primary trials was incomplete, so the absence of reported adverse events should not be interpreted as definitive evidence of safety. For instance, (Su et al., 2021) found no significant difference in the risk of adverse events between acupuncture groups and control groups. Peng et al. (2004) noted that no adverse events were reported in the included trials. The evidence quality for safety outcomes was consistently rated as Very Low, primarily due to imprecision (very wide confidence intervals) and the underreporting of adverse events in the primary studies.

### 3.4 Results of acupoint data mining

To elucidate the underlying treatment laws in the included studies, we performed a frequency analysis of the selected acupoints and meridians. As shown in Figure 5, the usage of meridians was unevenly distributed. The Governor Vessel (GV) was the most frequently selected meridian, accounting for a dominant 18.8% of the total usage. This was followed by the Bladder Meridian of Foot-Taiyang (BL) at 10.5% and the Stomach Meridian of Foot-Yangming (ST) at 9.8%. The Spleen Meridian (6.8%) and Kidney Meridian (6.0%) also occupied notable proportions.

**FIGURE 5:**
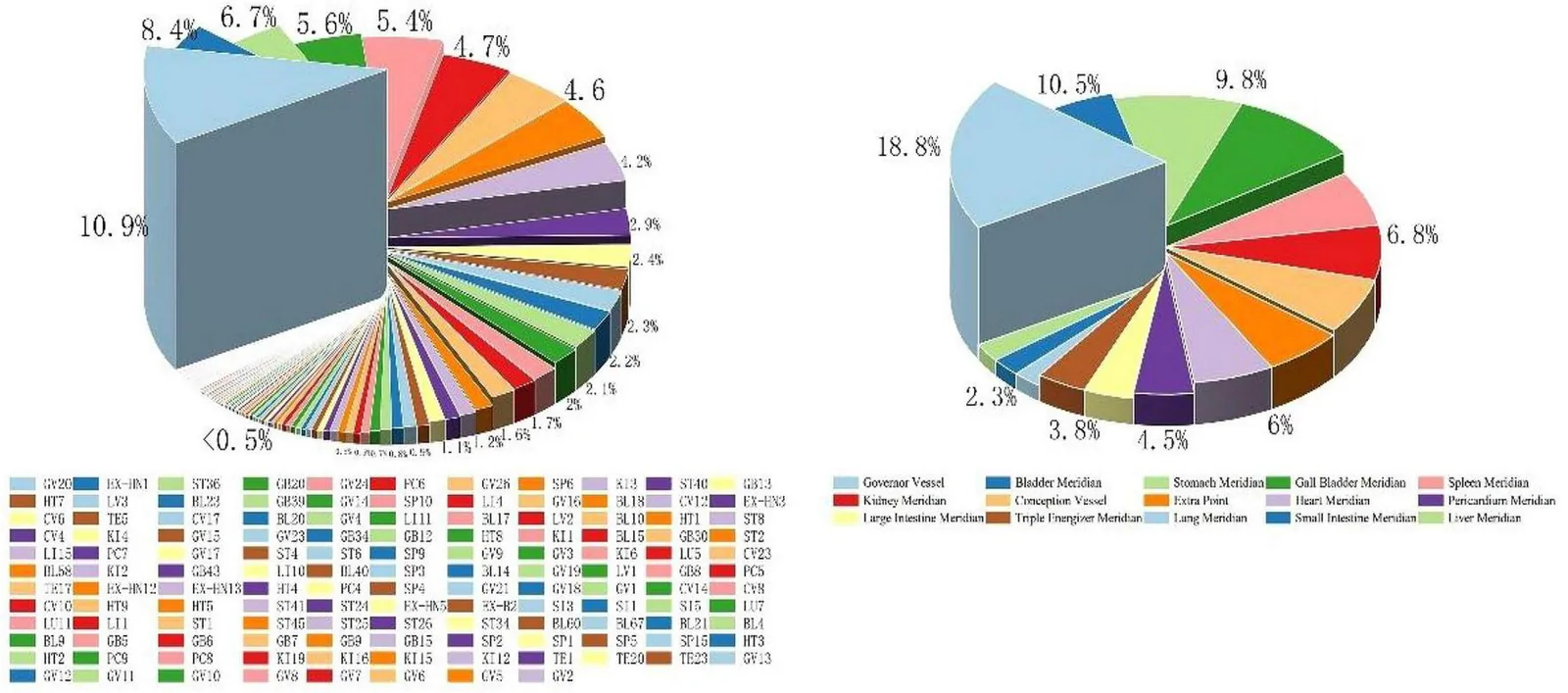
Visualization distribution of acupoint data mining.

Regarding specific acupoints, the analysis identified a cluster of high-frequency points. Baihui (GV20) was the most frequently used acupoint, representing 10.9% of the total frequency. Sishencong (EX-HN1) ranked second with 8.4%, followed by Zusanli (ST36) at 6.7%. Other frequently used points included Fengchi (GB20, 5.6%), Shenting (GV24, 5.4%), Neiguan (PC6, 4.7%), and Shuigou (GV26, 4.6%). The data indicates a preference for acupoints located on the head and face region, supplemented by distal points on the limbs.

## 4 Discussion

The pathogenesis of VD is relatively complex, primarily centered on cerebral ischemia and hypoxia due to cerebrovascular lesions, which subsequently lead to neuronal damage (Chen et al., 2024). Established pathogenic mechanisms involve several key processes. Impairment of the cholinergic system, resulting from reduced cerebral blood flow, is manifested as decreased acetylcholine synthesis, thereby affecting learning and memory functions (Chen et al., 2024; Dhaliwal et al., 2024; Xiang et al., 2022). Oxidative stress is characterized by an imbalance between the production and clearance of free radicals, leading to lipid peroxidation and neuronal structural damage (Chen et al., 2018; Yao et al., 2019). During cerebral ischemia, excessive glutamate release activates NMDA receptors, inducing calcium influx overload and ultimately resulting in neuronal necrosis, a process known as excitatory amino acid toxicity (Feldmann et al., 2019; Yu et al., 2023). Crucially, this cascade of ischemic insults disrupts activity-dependent synaptic plasticity and impairs long-term potentiation (LTP), which serves as the cellular basis for learning and memory. Recent neuroscientific evidence underscores that the fine-tuning of peri-synaptic extracellular matrix remodeling critically regulates the consolidation of LTP and hippocampal-dependent memory (Liu X. et al., 2025), highlighting a pivotal target for cognitive rescue. Furthermore, inflammatory responses play a significant role, as cerebral ischemia activates microglia to release factors such as tumor necrosis factor-α (TNF-α) and interleukin-1β (IL-1β), thereby exacerbating neuronal injury (Song et al., 2024; Tang, 2024). Additional factors contributing to the disease process include neuronal apoptosis, genetic determinants, and gut microbiota dysbiosis (Pang, 2019; Xu et al., 2017; Yao et al., 2019). Concurrently, the pathogenesis also involves multiple pathways (Feng et al., 2024; Niu et al., 2021; Wang et al., 2020). Preliminary research suggests that acupuncture may exert modulating effects across these multiple targets and pathways, such as potentially regulating cholinergic neurotransmitter levels, alleviating oxidative stress, and mitigating inflammatory responses (Jin et al., 2026; Li et al., 2022; Liu Q. et al., 2025; Wang, 2010; Zhang et al., 2021). However, given the current limitations in evidence certainty, these observed multi-target actions should be interpreted as potential signals of clinical benefit rather than established effectiveness. While these mechanisms offer a plausible theoretical framework for why acupuncture might support cognitive improvement and activities of daily living (Li et al., 2024; Zhao et al., 2024), they must be rigorously validated in higher-quality trials before definitive clinical recommendations can be formulated.

This study comprehensively evaluated and integrated evidence from 16 SRs/MAs on acupuncture for VD, aiming to offer more reliable evidence-based support for the application of acupuncture therapy in this field and to guide future clinical research.

The 16 SRs/MAs included in this study demonstrated a high degree of consistency in their conclusions, generally suggesting that acupuncture, either as a standalone therapy or in combination with conventional medication (Western or Chinese medicine), has a positive effect on improving cognitive function (e.g., MMSE, HDS scores), activities of daily living (ADL scores), and overall clinical efficacy in patients with VD. However, this optimistic conclusion must be interpreted with extreme caution, as our in-depth evaluation revealed widespread and serious deficiencies in the methodology, reporting standards, and evidence quality of these studies, significantly limiting the reliability and generalizability of the existing findings.

Regarding methodological quality, the results of the AMSTAR-2 assessment are concerning. Among the 16 SRs/MAs, the vast majority were rated as “low quality” or “critically low quality,” with only one rated as “high quality” and one as “moderate quality.” The core issues leading to the low ratings centered on several key items: (1) Lack of a pre-registered study protocol: Almost all studies failed to develop and register a protocol prior to execution (Item 2), substantially increasing the risk of selective reporting bias; for older reviews published before prospective registration (e.g., PROSPERO) became common practice, this may partly reflect an absence of reported registration rather than a complete lack of pre-specification; (2) Incomplete literature search: The search strategies of some studies were described as “partially meeting” or “not meeting” the criteria (Item 4), potentially omitting relevant studies; (3) Widespread absence of excluded study lists: Most studies did not provide a list of excluded literature with justifications (Item 7), reducing the transparency and credibility of the screening process. The prevalence of these critical flaws is the primary reason for the overall low methodological quality.

In terms of completeness of reporting, the results of the assessment against the PRISMA checklist revealed uneven reporting standards among the included studies. Reporting rates for several key items were extremely low, such as “Protocol and registration” (Item 24), “Study selection process” (Item 16), “Certainty of evidence” (Item 22), and “Funding” (Item 25), with most studies lacking information in these areas. Incomplete reporting not only compromises the transparency and reproducibility of the research but also makes it difficult for readers to fully assess the rigor of the study.

The most critical finding of this overview stems from the assessment of evidence quality. Using the GRADE system to rate the outcomes, we found that the vast majority of all evaluated outcomes were graded as “Low” or “Very Low” quality of evidence, with only one outcome from one study rated as “High” quality. The evidence was commonly downgraded for four main reasons: (1) Risk of bias (Limitations) – This was the primary downgrading factor, affecting almost all outcomes. It originated from the frequent issues of “inadequate allocation concealment” and “lack of blinding” in the original RCTs, leading to a high risk of performance and detection bias. (2) Inconsistency – Meta-analyses of several key outcomes, including MMSE and ADL, demonstrated substantial heterogeneity. However, most SRs/MAs did not explore its potential sources through subgroup or sensitivity analyses. Consequently, the stability of the pooled estimates remains uncertain. (3) Publication bias – Several outcomes were downgraded due to “funnel plot asymmetry” or other factors suggesting potential publication bias, indicating that studies with positive results might be more likely to be published. (4) Imprecision – Some outcomes were downgraded due to insufficient sample sizes or “wide confidence intervals,” reducing the precision of the results.

Furthermore, a critical methodological limitation identified across the included studies is the widespread reliance on composite outcome measures, particularly the “Total Effective Rate” and “Clinical Efficacy.” Although these indices have historically been favored in regional clinical research for providing a pragmatic overall assessment of treatment response, they lack internationally accepted definitions and standardization, thereby limiting their interpretability and comparability across studies. The “Total Effective Rate” typically combines multiple heterogeneous clinical indicators based on arbitrarily defined thresholds of improvement, which may obscure meaningful changes in individual outcome domains and increase the risk of detection bias, especially in studies where investigator blinding is difficult to implement.

To improve methodological rigor, facilitate cross-study comparisons, and promote international acceptance of acupuncture research in vascular dementia (VD), future clinical trials should move beyond these composite efficacy measures. Greater emphasis should be placed on validated, internationally recognized cognitive and functional outcome assessments, including the Mini-Mental State Examination (MMSE) and Montreal Cognitive Assessment (MoCA) for cognitive evaluation, as well as the Activities of Daily Living (ADL) scale, Barthel Index (BI), and Hasegawa Dementia Scale (HDS) for functional and daily living assessments. The adoption of these standardized and objective endpoints is essential for generating high-quality evidence, enhancing reproducibility, and ultimately supporting the incorporation of acupuncture into international clinical guidelines for stroke-related cognitive impairment and vascular dementia.

The methodological weaknesses identified in this overview are broadly consistent with those reported in other umbrella reviews of dementia interventions. However, several characteristics appear to be unique to acupuncture research. For instance, recent overviews evaluating acupuncture for Alzheimer’s disease or mild cognitive impairment similarly highlighted a pervasive lack of pre-registered protocols and identical downgrading under the GRADE system due to a high risk of bias in primary trials. This cross-study consistency underscores a systemic challenge in traditional alternative medicine research, where clinical trial execution often outpaces strict adherence to prospective systematic review registration. Compared with other non-pharmacological interventions for dementia, acupuncture presents a different pattern of methodological challenges. Behavioral interventions, such as physical exercise, cognitive training, and music therapy, are often affected by high attrition rates and performance bias because participant blinding is difficult. In contrast, heterogeneity in acupuncture research mainly arises from variations in stimulation parameters, acupoint selection, and control conditions. While non-pharmacological behavioral therapies struggle with standardized “dosage” delivery, acupuncture’s heterogeneity stems predominantly from the diverse parameters of needle stimulation, variations in acupoint selection, and the widespread absence of rigorous sham controls. Consequently, while behavioral evidence syntheses often suffer from high drop-out rates, acupuncture evidence syntheses are primarily compromised by a high risk of performance bias and inflated effect sizes driven by cultural context and lack of blinding, a critical insight that requires dedicated methodological rectifications in future neurodegenerative research.

To our knowledge, this study is among the first to systematically conduct an overview of SRs/MAs on acupuncture for VD utilizing multiple tools including AMSTAR-2, PRISMA, and GRADE. Through this multidimensional assessment, the study not only synthesized the existing evidence but, more importantly, highlighted common methodological shortcomings in the field, providing clear guidance for improving the quality of future research. Additionally, from a methodological perspective, this overview rigorously quantified the overlap of primary studies among the included systematic reviews by calculating the CCA. The resulting CCA value of 2.09% falls within the range of slight overlap. This indicates that the 16 synthesized reviews relied on largely distinct pools of primary trials, which reduces, but does not eliminate, concerns regarding redundancy and double-counting of primary trial data. Low overlap does not by itself demonstrate independence of patient populations, research groups, treatment protocols, or sources of bias.

Crucially, the evaluated comparisons address distinct causal questions: comparisons of acupuncture plus conventional therapy versus conventional therapy alone measure incremental add-on benefits, whereas comparisons against active drugs reflect relative pragmatic performance. Because very few of the included trials used sham-acupuncture controls, the pooled estimates cannot isolate specific needling effects from non-specific contextual and placebo responses. These distinct comparator types should therefore not be interpreted as equivalent evidence of acupuncture-specific efficacy.

These primary-trial weaknesses do not merely qualify our conclusions as a closing caveat; they directly govern how all of the apparent treatment effects must be interpreted. The pervasive risk of performance and detection bias arising from unblinded designs, combined with small-study effects, means that the observed pooled benefits may substantially overestimate the true specific effect of acupuncture.

Importantly, statistical significance in the pooled estimates must not be equated automatically with clinically meaningful benefit. The pooled cognitive estimates in the included reviews were heterogeneous in both magnitude and metric: reported mean differences for the MMSE ranged from 0.87 to 5.06 points, whereas other estimates were expressed as standardized mean differences (SMD 0.39–1.09) or, in one case, as a risk ratio, and therefore cannot be placed on a common point scale. More importantly, validated minimal clinically important differences (MCIDs) specific to vascular dementia have not been established for the MMSE, HDS, MoCA, or ADL. Thresholds proposed in other dementia populations (for example, Alzheimer’s disease) cannot be assumed to apply to a mixed VD and VCI population that differs in baseline severity, disease stage, and duration of follow-up, and estimates expressed as standardized mean differences have no direct MCID interpretation. We therefore deliberately refrain from applying any generic MCID threshold and interpret the pooled results only in terms of the direction of effect together with the certainty of evidence (GRADE). Accordingly, statistically significant superiority over control interventions should not be read as proven, clinically noticeable benefit, and establishing VD-specific MCIDs should be a priority for future research.

### 4.1 Implications for future research

For primary clinical research (RCTs), future studies should strictly adhere to the CONSORT statement by designing and implementing large-sample, multicenter, long-duration randomized controlled trials. Key focus areas include: (1) Employing appropriate methods for random sequence generation and allocation concealment; (2) Implementing blinding of participants, outcome assessors, and, where feasible, practitioners; (3) Adopting internationally recognized core outcome sets for dementia research and conducting long-term follow-up. Only three of the included SRs/MAs reported adverse events, and even these were limited by severe underreporting and very low-certainty evidence. The absence of reported adverse events therefore constitutes absence of evidence rather than evidence of safety, and the safety profile of acupuncture for VD remains undetermined pending standardized adverse-event ascertainment. Therefore, future RCTs should incorporate standardized procedures for monitoring, classifying, and reporting acupuncture-related adverse events (such as subcutaneous hematoma, fainting during acupuncture, or local pain) to establish a transparent and reliable safety database.

For secondary research (SRs/MAs), researchers should follow the PRISMA statement. A study protocol should be registered on platforms like PROSPERO before commencing the review. Literature searches must be comprehensive and transparent, with full search strategies and lists of excluded studies provided. During data analysis, heterogeneity should be addressed judiciously, and the robustness of the results should be evaluated. Furthermore, both primary and secondary studies should provide detailed reports of the specific components of the acupuncture treatment protocol, including: rationale for acupoint selection, for which data-mining and knowledge-graph analyses of acupuncture research in vascular conditions provide a structured basis (Che and Cheng, 2023), needling techniques (e.g., lifting-thrusting, twirling), stimulation parameters (e.g., frequency and waveform for electroacupuncture), deqi sensation, treatment frequency, treatment course, as well as the qualifications and experience of the practitioners. Standardized and transparent reporting of protocols is crucial for ensuring the reproducibility of studies and the comparability of their results.

### 4.2 Implications for clinical practice

The findings of this overview have significant implications for bridging the gap between current clinical evidence and guideline recommendations for VD. Currently, a notable discrepancy exists regarding the status of acupuncture in clinical guidelines. While acupuncture is recommended in several authoritative Chinese guidelines–such as the Chinese Guidelines for the Diagnosis and Treatment of Vascular Cognitive Impairment (2024 Edition) (Chinese Stroke Association Vascular Cognitive Impairment Subcommittee, 2024) and the Chinese Stroke Association Guidelines for Clinical Management of Cerebrovascular Diseases (Second Edition) (Chinese Stroke Association, 2023) –as an effective adjuvant therapy, it has not yet been incorporated into mainstream international guidelines. Major international consensus statements, such as the scientific statement from the American Heart Association/American Stroke Association (AHA/ASA; Gorelick et al., 2011) and the JACC scientific expert panel (Iadecola et al., 2019), primarily focus on vascular risk factor management and do not currently recommend acupuncture as a standard intervention. This exclusion is largely attributed to the lack of standardized treatment protocols and high-quality evidence in previous years. However, while the consistent direction of effect across studies provides a signal of potential clinical benefit, the overwhelmingly low certainty of the evidence indicates that current data are insufficient to support routine clinical recommendations in international guidelines. Present evidence supports prioritizing acupuncture for further high-quality research rather than immediate guideline adoption.

To address the issue of protocol heterogeneity that hinders international guideline inclusion, our data mining results offer a clear, evidence-based framework for standardization. The analysis revealed an overwhelming predominance of the Governor Vessel (GV), which accounted for 18.8% of total meridian usage. Based on this, we propose a consensus-based reference “Governor Vessel-regulating” protocol as a candidate reference pattern that reflects commonly used clinical practice and merits prospective evaluation. This strategy is supported by both Traditional Chinese Medicine (TCM) theory–where the GV “enters the brain”–and emerging high-quality evidence. A recent meta-analysis published in Ageing Research Reviews by Han et al. (2024) confirmed that Governor Vessel acupuncture therapy significantly improves cognitive function and daily living activities in post-stroke cognitive impairment, a condition sharing key pathological mechanisms with VD. These findings have been interpreted as suggesting that stimulating GV acupoints may modulate cerebral perfusion and synaptic neuroplasticity; however, such mechanisms remain external hypotheses that warrant direct biomarker investigation in VD cohorts (Han et al., 2024). Importantly, while the acupoint selection frequencies obtained through data mining primarily reflect clinical popularity and consensus among practitioners, this consensus-based reference protocol (specifically GV20, EX-HN1, and ST36) reflects common clinical practice and practitioner consensus rather than established comparative effectiveness. Because the underlying analysis establishes frequency of use rather than comparative efficacy, and the source evidence is predominantly of low or very low certainty, this combination is best regarded as a frequently observed candidate reference protocol. Rather than establishing superior effectiveness, it offers a standardized, hypothesis-generating baseline that merits prospective testing in future rigorously designed, sham-controlled trials.

Based on the high-frequency acupoint clusters identified, we recommend a consensus-based reference acupoint combination for prospective testing in future RCTs that follows a “Local (Cranial) + Distal (Limb)” approach. Specifically, the core cranial module should prioritize GV20 (Baihui) and EX-HN1 (Sishencong), which accounted for 10.9% and 8.4% of usage, respectively, to “awaken the mind” through local stimulation. This should be integrated with the systemic support module using ST36 (Zusanli), the key point of the Stomach Meridian, to “fortify the spleen and replenish qi,” addressing the systemic deficits often present in elderly VD patients. Prospective evaluation of this standardized candidate protocol could help reduce treatment variability in future trials. Accordingly, researchers should test this frequently used acupoint combination in future large-scale, multicenter, sham-controlled RCTs rather than adopting it as an established prescription. Generating high-quality evidence using standardized protocols may help clarify the role of acupuncture in future international guidelines for vascular dementia.

## 5 Limitations

Although this study demonstrates innovation in topic selection and evidence synthesis, the reliability of its conclusions remains constrained by several unavoidable limitations, primarily stemming from the methodological and reporting deficiencies of the included systematic reviews and their underlying original studies. One concern is the potential for publication and language bias. All 16 included systematic reviews were conducted by Chinese researchers, and the original studies they incorporated were predominantly performed and published in China. Although our search covered Chinese and English databases, relevant studies published in other languages (e.g., Japanese, Korean, German) or indexed in regional databases (e.g., J-STAGE, KCI) may have been missed. This geographical homogeneity introduces crucial implications for the generalizability (external validity) of our findings. The pooled clinical outcomes may be tightly coupled with specific genetic backgrounds, dietary habits, and healthcare delivery models unique to the Chinese demographic. Furthermore, since acupuncture is deeply rooted in Chinese culture, patients in these trials likely exhibit high baseline familiarity and psychological acceptance of the therapy. Such cultural alignment can potentially amplify non-specific contextual effects, patient expectations, and placebo responses, which may not be replicable in Western countries or populations where acupuncture is perceived as an unfamiliar alternative. Consequently, extrapolating these conclusions to non-Chinese contexts requires caution, and multinational, multi-center trials are highly warranted. Another limitation arises from the significant clinical and methodological heterogeneity present. This is a core issue undermining the reliability of evidence in this field. Clinically, diagnostic criteria varied across studies, and there was a lack of uniform definition for the severity or subtypes of VD. This resulted in considerable clinical variation, weakening the comparability between studies and the validity of pooled estimates. Methodological heterogeneity was even more pronounced. The included reviews covered various interventions; for example, (Xue et al., 2024) focused on acupuncture combined with Chinese herbal medicine, whereas W, T 2017 (Wan et al., 2017) evaluated electroacupuncture alone. Furthermore, the original studies differed substantially in treatment protocols, including stimulation intensity, acupoint selection, treatment frequency, and duration. The lack of standardization in these procedural details, along with variations in practitioner skill, further exacerbated methodological differences, affecting the consistency of outcome assessments. Furthermore, there is a widespread lack of placebo controls, representing a key methodological shortcoming. Notably, the vast majority of original studies included in these systematic reviews did not employ rigorous sham acupuncture or placebo controls. Many studies, such as those incorporated in Lan et al. (2020) and Chen (2016), used conventional Western drug therapy as the control rather than a placebo. This makes it difficult to distinguish between the specific therapeutic effects of acupuncture and potent non-specific effects (e.g., placebo response, patient expectation, practitioner-patient interaction), potentially leading to an overestimation of acupuncture’s true efficacy. Additionally, inadequate implementation of randomization, allocation concealment, and blinding poses significant problems. These issues seriously compromise the internal validity of the primary studies. Our GRADE evidence profile clearly shows that the evidence level for nearly all outcomes was downgraded, mainly due to a high risk of bias in the original studies, particularly from insufficient reporting of randomization and allocation concealment. If researchers or participants were aware of treatment allocation at the trial’s outset, it could introduce selection bias and undermine group comparability. Moreover, blinding practitioners and participants is especially challenging in acupuncture trials, increasing the risk of performance and detection bias. These methodological flaws weaken the internal validity of the primary studies and further reduce our confidence in their conclusions. Finally, small sample sizes and insufficient statistical power constrain the robustness of the analysis. The statistical power was limited by the overall sample size and the small scale of individual trials. Many included meta-analyses were based on a small number of trials, which often had fewer than 50 participants per group. For instance, the analysis of BBS scores in Yan et al. (2024) was based on only two trials with a total of 124 participants. Small studies are more susceptible to random error and tend to report inflated treatment effects that may not be replicable–a phenomenon known as the small-study effect. Limited statistical power also increases the risk of Type II error, potentially masking real but modest treatment effects. In the GRADE assessments, many outcomes were downgraded for imprecision due to wide confidence intervals, a direct consequence of insufficient sample sizes.

## 6 Conclusion

In conclusion, although acupuncture shows potential signals of benefit in improving cognitive function and activities of daily living for VD, the clinical evidence remains inconclusive. This uncertainty is primarily due to the low or very low quality of current evidence, which is limited by methodological weaknesses, imprecision, and heterogeneity in the primary trials. Further rigorous, large-scale studies are required to establish definitive conclusions.

## Data Availability

The original contributions presented in this study are included in this article/Supplementary material, further inquiries can be directed to the corresponding authors.

## Supplementary material

SUPPLEMENTARY FILE 1 Search strategy for PubMed (search steps #1–#10; 32 records retrieved).

SUPPLEMENTARY FILE 2 Study-by-review citation matrix for the assessment of primary studyoverlap. Rows list the 242 unique primary RCTs and columns the 16included SRs/MAs; a filled cell indicates that the primary study is included inthe corresponding review. Summing the per-review trial counts givesN D 318 primary-study occurrences, and the corrected covered area (CCA)is 2.09% (m D 16, N D 318, r D 242).
