# Supplementary material for: Acupuncture for vascular dementia: an overview of systematic reviews and meta-analysis
Source: Front Neurosci. 2026 Oct 19;20:1796611. doi: 10.3389/fnins.2026.1796611 (PMC13724493; doi:10.3389/fnins.2026.1796611)
Supplement: Supplementary file 1 [file Supplementary_file_1.docx]

Supplementary 1

| **NO** | **Search Details** | **Results** |
| --- | --- | --- |
| #1 | "Dementia, Vascular"[Mesh] | 8,074 |
| #2 | ((((((((((((((((((((((((((((((((((((((Dementias, Vascular) OR (Vascular Dementias)) OR (Vascular Dementia)) OR (Arteriosclerotic Dementia)) OR (Arteriosclerotic Dementias)) OR (Dementia, Arteriosclerotic)) OR (Dementias, Arteriosclerotic)) OR (Binswanger Disease)) OR (Disease, Binswanger)) OR (Encephalopathy, Binswanger)) OR (Chronic Progressive Subcortical Encephalopathy)) OR (Binswanger Encephalopathy)) OR (Leukoencephalopathy, Subcortical)) OR (Leukoencephalopathies, Subcortical)) OR (Subcortical Leukoencephalopathies)) OR (Encephalopathy, Subcortical Arteriosclerotic)) OR (Encephalopathy, Chronic Progressive Subcortical)) OR (Encephalopathy, Subcortical, Chronic Progressive)) OR (Subcortical Encephalopathy, Chronic Progressive)) OR (Subcortical Leukoencephalopathy)) OR (Subcortical Arteriosclerotic Encephalopathy)) OR (Arteriosclerotic Encephalopathy, Subcortical)) OR (Arteriosclerotic Encephalopathies, Subcortical)) OR (Encephalopathies, Subcortical Arteriosclerotic)) OR (Subcortical Arteriosclerotic Encephalopathies)) OR (Encephalopathy, Binswanger's)) OR (Binswanger's Encephalopathy)) OR (Encephalopathy, Binswangers)) OR (Binswanger's Disease)) OR (Binswangers Disease)) OR (Disease, Binswanger's)) OR (Subcortical Vascular Dementia)) OR (Dementias, Subcortical Vascular)) OR (Dementia, Subcortical Vascular)) OR (Subcortical Vascular Dementias)) OR (Vascular Dementias, Subcortical)) OR (Vascular Dementia, Subcortical)) OR (Vascular Dementia, Acute Onset)) OR (Acute Onset Vascular Dementia) | 22,881 |
| #3 | ("Dementia, Vascular"[Mesh]) OR (((((((((((((((((((((((((((((((((((((((Dementias, Vascular) OR (Vascular Dementias)) OR (Vascular Dementia)) OR (Arteriosclerotic Dementia)) OR (Arteriosclerotic Dementias)) OR (Dementia, Arteriosclerotic)) OR (Dementias, Arteriosclerotic)) OR (Binswanger Disease)) OR (Disease, Binswanger)) OR (Encephalopathy, Binswanger)) OR (Chronic Progressive Subcortical Encephalopathy)) OR (Binswanger Encephalopathy)) OR (Leukoencephalopathy, Subcortical)) OR (Leukoencephalopathies, Subcortical)) OR (Subcortical Leukoencephalopathies)) OR (Encephalopathy, Subcortical Arteriosclerotic)) OR (Encephalopathy, Chronic Progressive Subcortical)) OR (Encephalopathy, Subcortical, Chronic Progressive)) OR (Subcortical Encephalopathy, Chronic Progressive)) OR (Subcortical Leukoencephalopathy)) OR (Subcortical Arteriosclerotic Encephalopathy)) OR (Arteriosclerotic Encephalopathy, Subcortical)) OR (Arteriosclerotic Encephalopathies, Subcortical)) OR (Encephalopathies, Subcortical Arteriosclerotic)) OR (Subcortical Arteriosclerotic Encephalopathies)) OR (Encephalopathy, Binswanger's)) OR (Binswanger's Encephalopathy)) OR (Encephalopathy, Binswangers)) OR (Binswanger's Disease)) OR (Binswangers Disease)) OR (Disease, Binswanger's)) OR (Subcortical Vascular Dementia)) OR (Dementias, Subcortical Vascular)) OR (Dementia, Subcortical Vascular)) OR (Subcortical Vascular Dementias)) OR (Vascular Dementias, Subcortical)) OR (Vascular Dementia, Subcortical)) OR (Vascular Dementia, Acute Onset)) OR (Acute Onset Vascular Dementia)) | 22,881 |
| #4 | "Acupuncture"[Mesh] OR "Acupuncture Therapy"[Mesh] OR "Acupuncture, Ear"[Mesh] OR "Acupuncture Points"[Mesh] | 32,996 |
| #5 | ((((((((((((((((((((((((((Acupuncture) OR (Acupuncture Therapy)) OR (Acupuncture, Ear)) OR (Acupuncture Points)) OR (Pharmacopuncture)) OR (Acupuncture Treatment)) OR (Acupuncture Treatments)) OR (Treatment, Acupuncture)) OR (Therapy, Acupuncture)) OR (Pharmacoacupuncture Treatment)) OR (Treatment, Pharmacoacupuncture)) OR (Pharmacoacupuncture Therapy)) OR (Therapy, Pharmacoacupuncture)) OR (Acupotomy)) OR (Acupotomies)) OR (Acupunctures, Ear)) OR (Ear Acupunctures)) OR (Acupuncture, Auricular)) OR (Acupunctures, Auricular)) OR (Auricular Acupunctures)) OR (Auricular Acupuncture)) OR (Ear Acupuncture)) OR (Acupuncture Point)) OR (Point, Acupuncture)) OR (Points, Acupuncture)) OR (Acupoints)) OR (Acupoint) | 51,287 |
| #6 | ("Acupuncture"[Mesh] OR "Acupuncture Therapy"[Mesh] OR "Acupuncture, Ear"[Mesh] OR "Acupuncture Points"[Mesh]) OR (((((((((((((((((((((((((((Acupuncture) OR (Acupuncture Therapy)) OR (Acupuncture, Ear)) OR (Acupuncture Points)) OR (Pharmacopuncture)) OR (Acupuncture Treatment)) OR (Acupuncture Treatments)) OR (Treatment, Acupuncture)) OR (Therapy, Acupuncture)) OR (Pharmacoacupuncture Treatment)) OR (Treatment, Pharmacoacupuncture)) OR (Pharmacoacupuncture Therapy)) OR (Therapy, Pharmacoacupuncture)) OR (Acupotomy)) OR (Acupotomies)) OR (Acupunctures, Ear)) OR (Ear Acupunctures)) OR (Acupuncture, Auricular)) OR (Acupunctures, Auricular)) OR (Auricular Acupunctures)) OR (Auricular Acupuncture)) OR (Ear Acupuncture)) OR (Acupuncture Point)) OR (Point, Acupuncture)) OR (Points, Acupuncture)) OR (Acupoints)) OR (Acupoint)) | 51,287 |
| #7 | ("Meta-Analysis" [Publication Type] OR "Meta-Analysis as Topic"[Mesh] OR "Network Meta-Analysis" [Publication Type] OR "Network Meta-Analysis as Topic"[Mesh]) OR ("Systematic Review" [Publication Type] OR "Systematic Reviews as Topic"[Mesh]) | 424,141 |
| #8 | (((((((((((Meta Analysis as Topic) OR (Clinical Trial Overviews)) OR (Clinical Trial Overview)) OR (Overview, Clinical Trial)) OR (Overviews, Clinical Trial)) OR (Mixed Treatment Meta-Analysis)) OR (Multiple Treatment Comparison Meta-Analysis)) OR (Review, Systematic)) OR (Umbrella Review)) OR (Reviews Systematic as Topic)) OR (Systematic Review as Topic)) OR (Umbrella Reviews as Topic) | 453,866 |
| #9 | (("Meta-Analysis" [Publication Type] OR "Meta-Analysis as Topic"[Mesh] OR "Network Meta-Analysis" [Publication Type] OR "Network Meta-Analysis as Topic"[Mesh]) OR ("Systematic Review" [Publication Type] OR "Systematic Reviews as Topic"[Mesh])) OR ((((((((((((Meta Analysis as Topic) OR (Clinical Trial Overviews)) OR (Clinical Trial Overview)) OR (Overview, Clinical Trial)) OR (Overviews, Clinical Trial)) OR (Mixed Treatment Meta-Analysis)) OR (Multiple Treatment Comparison Meta-Analysis)) OR (Review, Systematic)) OR (Umbrella Review)) OR (Reviews Systematic as Topic)) OR (Systematic Review as Topic)) OR (Umbrella Reviews as Topic)) | 453,866 |
| #10 | ((("Dementia, Vascular"[Mesh]) OR (((((((((((((((((((((((((((((((((((((((Dementias, Vascular) OR (Vascular Dementias)) OR (Vascular Dementia)) OR (Arteriosclerotic Dementia)) OR (Arteriosclerotic Dementias)) OR (Dementia, Arteriosclerotic)) OR (Dementias, Arteriosclerotic)) OR (Binswanger Disease)) OR (Disease, Binswanger)) OR (Encephalopathy, Binswanger)) OR (Chronic Progressive Subcortical Encephalopathy)) OR (Binswanger Encephalopathy)) OR (Leukoencephalopathy, Subcortical)) OR (Leukoencephalopathies, Subcortical)) OR (Subcortical Leukoencephalopathies)) OR (Encephalopathy, Subcortical Arteriosclerotic)) OR (Encephalopathy, Chronic Progressive Subcortical)) OR (Encephalopathy, Subcortical, Chronic Progressive)) OR (Subcortical Encephalopathy, Chronic Progressive)) OR (Subcortical Leukoencephalopathy)) OR (Subcortical Arteriosclerotic Encephalopathy)) OR (Arteriosclerotic Encephalopathy, Subcortical)) OR (Arteriosclerotic Encephalopathies, Subcortical)) OR (Encephalopathies, Subcortical Arteriosclerotic)) OR (Subcortical Arteriosclerotic Encephalopathies)) OR (Encephalopathy, Binswanger's)) OR (Binswanger's Encephalopathy)) OR (Encephalopathy, Binswangers)) OR (Binswanger's Disease)) OR (Binswangers Disease)) OR (Disease, Binswanger's)) OR (Subcortical Vascular Dementia)) OR (Dementias, Subcortical Vascular)) OR (Dementia, Subcortical Vascular)) OR (Subcortical Vascular Dementias)) OR (Vascular Dementias, Subcortical)) OR (Vascular Dementia, Subcortical)) OR (Vascular Dementia, Acute Onset)) OR (Acute Onset Vascular Dementia))) AND (("Acupuncture"[Mesh] OR "Acupuncture Therapy"[Mesh] OR "Acupuncture, Ear"[Mesh] OR "Acupuncture Points"[Mesh]) OR (((((((((((((((((((((((((((Acupuncture) OR (Acupuncture Therapy)) OR (Acupuncture, Ear)) OR (Acupuncture Points)) OR (Pharmacopuncture)) OR (Acupuncture Treatment)) OR (Acupuncture Treatments)) OR (Treatment, Acupuncture)) OR (Therapy, Acupuncture)) OR (Pharmacoacupuncture Treatment)) OR (Treatment, Pharmacoacupuncture)) OR (Pharmacoacupuncture Therapy)) OR (Therapy, Pharmacoacupuncture)) OR (Acupotomy)) OR (Acupotomies)) OR (Acupunctures, Ear)) OR (Ear Acupunctures)) OR (Acupuncture, Auricular)) OR (Acupunctures, Auricular)) OR (Auricular Acupunctures)) OR (Auricular Acupuncture)) OR (Ear Acupuncture)) OR (Acupuncture Point)) OR (Point, Acupuncture)) OR (Points, Acupuncture)) OR (Acupoints)) OR (Acupoint)))) AND ((("Meta-Analysis" [Publication Type] OR "Meta-Analysis as Topic"[Mesh] OR "Network Meta-Analysis" [Publication Type] OR "Network Meta-Analysis as Topic"[Mesh]) OR ("Systematic Review" [Publication Type] OR "Systematic Reviews as Topic"[Mesh])) OR ((((((((((((Meta Analysis as Topic) OR (Clinical Trial Overviews)) OR (Clinical Trial Overview)) OR (Overview, Clinical Trial)) OR (Overviews, Clinical Trial)) OR (Mixed Treatment Meta-Analysis)) OR (Multiple Treatment Comparison Meta-Analysis)) OR (Review, Systematic)) OR (Umbrella Review)) OR (Reviews Systematic as Topic)) OR (Systematic Review as Topic)) OR (Umbrella Reviews as Topic))) | 32 |
